# Highly reliable LC-MS lipidomics database for efficient human plasma profiling based on NIST SRM 1950^1^

**DOI:** 10.1016/j.jlr.2024.100671

**Published:** 2024-10-10

**Authors:** Sara Martínez, Miguel Fernández-García, Sara Londoño-Osorio, Coral Barbas, Ana Gradillas

**Affiliations:** 1 Centro de Metabolómica y Bioanálisis (CEMBIO), Facultad de Farmacia, Universidad San Pablo-CEU, CEU Universities, Madrid, Spain; 2 Departamento de Ciencias Médicas Básicas, Facultad de Medicina, Universidad San Pablo-CEU, CEU Universities, Madrid, Spain

**Keywords:** glycerophospholipids, glycerolipids, sphingolipids, human plasma lipidome, in-house database, NIST SRM 1950, lipidomic profiling, lipid annotation, adduct profile

## Abstract

Liquid chromatography coupled to high-resolution mass spectrometry (LC-HRMS)-based methods have become the gold standard methodology for the comprehensive profiling of the human plasma lipidome. However, both the complexity of lipid chemistry and LC-HRMS-associated data pose challenges to the characterization of this biological matrix. In accordance with the current consensus of quality requirements for LC-HRMS lipidomics data, we aimed to characterize the NIST® Standard Reference Material for Human Plasma (SRM 1950) using an LC-ESI(+/−)-MS method compatible with high-throughput lipidome profiling. We generated a highly curated lipid database with increased coverage, quality, and consistency, including additional quality assurance procedures involving adduct formation, within-method *m/z* evaluation, retention behavior of species within lipid chain isomers, and expert-driven resolution of isomeric and isobaric interferences. As a proof-of-concept, we showed the utility of our in-house LC-MS lipidomic database —consisting of 592 lipid entries— for the fast, comprehensive, and reliable lipidomic profiling of the human plasma from healthy human volunteers. We are confident that the implementation of this robust resource and methodology will have a significant impact by reducing data redundancy and the current delays and bottlenecks in untargeted plasma lipidomic studies.

The lipidome, a structurally complex and diverse biological subsystem encompassing all lipids in a biological sample, stands as a key focal subject for biological and clinical research (1, 2, 3, 4). Holistic and comprehensive studies of the lipidome at the molecular species level require the use of advanced analytical techniques, from which liquid-chromatography coupled to high resolution mass spectrometry (LC-HRMS) has become the gold standard methodology (5, 6, 7). However, within the untargeted LC-HRMS-based lipidomic workflow, two major stages critically influence the overall coverage and quality of the lipidomic experiments: data processing and lipid identification (8, 9). In response to the highly extensive and complex raw data generated, lipidomic research has strategically relied on computational methodologies of increasing sophistication, such as specialized algorithms and tools (10, 11, 12, 13, 14). While computational solutions assisting data processing are indisputably valuable to support any omics analysis, it is a common practice in lipidomic studies to only annotate the subset of lipids deemed significant after statistical analyses (9). However, this approach introduces (i) statistical bias arising from the conservation of redundant unknown compounds and (ii) interpretative bias associated with the inability to contextualize relevant lipids within the global lipidome data. These disadvantages have been partially circumvented by the implementation of software-assisted automatic lipid annotation within the LC-HRMS–based lipidomics workflow before statistical analysis, achieving a higher number of lipid annotations (10, 11, 12, 13, 14).

The validation of lipid annotations provided by software tools requires manual and expert-driven evaluation of MS1 and tandem mass spectra (MS/MS or MS2) information to minimize false-positive annotation rates while addressing the issue of signal redundancy (15). In addition, manual evaluation is required to maximize lipid coverage through the assessment of low-level and/or low-quality lipid signals, as these cannot be effectively managed by the current state-of-the-art software (16). The comprehensive assessment of confidence in the annotation of lipids requires the manual evaluation of all complementary pieces of information. This process is particularly time-consuming (17) as key areas —requiring expert knowledge— must be addressed, (Table 1).

**Table 1:** Expert knowledge for comprehensive LC-MS data interpretation

MS1 spectra interpretation under electrospray ionization (ESI)		Ref.
i	The formation of adducts and cluster by mobile phase and/or additives.	(18, 19)
ii	The in-source fragmentation (ISF) of some lipids producing unintentional fragment ions and the natural occurrence of ion source–related artifacts.	(20, 21, 22)

**Discrimination of isobaric and isomeric lipids**		

iii	The high isomeric and isobaric complexity present in the lipidome and the potential overlaps and interferences.	(23)

**RP-chromatographic separation: elution profile of lipids**		

iv	The relationship between lipid structure and reversed-phase (RP) chromatographic retention behavior (https://www.agilent.com/cs/library/applications/5991-8042EN.pdf).	(24)

**MS/MS spectra interpretation**		

v	The lipid fragmentation pathways, and the mechanisms involved, leading to the generation of structurally related characteristic fragment ions.	(25)
vi	The low-quality of many automated-MS/MS spectra leading to convoluted spectra.	(26, 27)

The need for accurate and fast lipid annotation prior to statistical analysis has promoted during the last decade the generation of curated, in-house databases containing multiple orthogonal (i.e., independent) pieces of information as comprehensive resources for lipid identification (28, 29). Information within these lipid databases is often not only restricted to MS/MS-associated lipid spectra libraries (30) but can also contain accurate mass values (mass to charge, *m/z*), retention times (RT) (24) and molecular formula-associated isotopic patterns, and occasionally collision cross-section values (31), as useful data for lipid identification (29). The use of such tailored, highly curated, matrix-specific databases for targeted data reprocessing has become a solution to tackle the challenges associated with lipidomics data reprocessing and annotation (29); these databases have steadily grown in lipid molecular species coverage and have increased our annotation efficiency while decreasing the time required for manual curation (32). This ultimately aims to provide maximum coverage and true positive annotation rates while minimizing false positive annotation rates, data redundancy, and data processing time.

Along with the advances in analytical performance and community knowledge on MS-based lipidomics research, the choice of appropriate reference materials (RM) becomes crucial for enhancing data quality and standardization across studies and laboratories (33). For this purpose, lipid databases generated from RM data offer a valuable resource. In particular, plasma RM stand out as representative for this metabolite-rich biofluid, which is acquired through minimally invasive procedures and has extensive applications in clinical practice. Specifically, the certified NIST® SRM® 1950-Metabolites in Frozen Human Plasma (SRM 1950) has been widely adopted by the metabolomics community due to its established and certified values for a large number of metabolites (33). Although the SRM 1950 polar metabolome has been extensively characterized, its reference lipidome profiling is comparatively less detailed (33). Harmonizing studies have been performed but often provide the sum composition of lipid species, thus providing reduced information on the lipid-bound fatty acyl/alkyl/alkenyl chain composition of SRM 1950 (34, 35). Therefore, improving the coverage, specificity, confidence, and annotation level of SRM 1950 lipid annotation could yield benefits by providing a more comprehensive and realistic overview of the plasma lipidome. Significant untargeted studies of SRM 1950 have already been reported contributing to the lipidome characterization of this RM (29, 34, 36, 37) with variations in the results and annotation information based on the methodologies applied (supplemental Table S1).

For construction of lipid databases —using MS/MS-based experiments for lipid characterization— classic data-dependent acquisition (DDA) fragmentation methods still lead the way in lipidomics (supplemental Table S1A, (38)). Unlike data-independent acquisition (DIA), DDA —specifically when iterative exclusion DDA experiments is applied (36, 18)— offers the possibility to obtain real and selective MS/MS spectra and thus to improve lipid assignment when essential technical parameters are optimized to ensure maximum performance (39). Although different DIA approaches —including sequential precursor ion fragmentation (MS/MS^ALL^ which can be categorized into two methods: MS^E^ and all-ion fragmentation (29)); sequential window acquisition of all theoretical mass spectra, and an innovative mode of acquisition known as scanning quadrupole DIA (SONAR) (40)— have improved the lipid coverage by facilitating the annotation of compounds associated with low-abundance precursor ions, they use a much wider precursor-ion isolation window. As a result, much higher complexity and contamination of MS/MS spectra is observed that must be deconvoluted making data processing more challenging (40, 41).

In addition, while the annotation coverage in the SRM 1950 lipid databases reported is around 500 lipid species on average (supplemental Table S1A), they often lack the reliable criteria according to current state-of-the-art quality assurance procedures to ensure annotation at the highest possible level (17). This involves a detailed evaluation of systematic criteria, within the method —such as *m/z* values, adduct profiles, the generation of in-source fragments and neutral losses (NL), RP chromatographic behavior, and structure-related diagnostic ions— all of which is only partly addressed (Table 1).

In our study, we aim to enhance the existing results by using extensive experimental data curation procedures, thus providing a more refined annotation confidence and consistency compared to literature data and achieving a more comprehensive understanding of the lipid profile of SRM 1950 (29, 34, 36, 37).

Here, as a result of our pipeline for the annotation of lipid species, we have built an increased-coverage LC-MS in-house lipid database composed of 592 lipid species present in SRM 1950 —including lipid classification, name, molecular formula, RT (min), *m/z*, relative abundances, and an expanded characterization of the profile of associated adducts and their hierarchy— using reversed-phase ultra-high performance liquid chromatography (RP-UHPLC) coupled to a quadrupole-time-of-flight mass spectrometer (QTOF-MS) for lipid profiling (supplemental Table S2).

In parallel to the construction of the database, an in-depth manual examination of the MS1 and MS/MS data and a thorough quality assessment of all available orthogonal information was carried out for the systematization of the downstream data curation pipeline. Such a rigorous inspection process, although time-consuming, was a crucial step to validate the LC-HRMS experimental data, which ensured data quality, high confidence, and integrity of lipid annotation and lipidome coverage.

Finally, our in-house LC-MS lipid database allowed us to overcome the challenge of the untargeted data pre-processing step by performing an MS1 database matching targeted peak extraction approach from accurate mass, molecular formula, and RT data, using an algorithm from proprietary vendor-specific software (Agilent MassHunter Profinder). Our strategy was successfully applied to human plasma samples from ten healthy volunteers and effectively removed redundancy, reduced processing time, and selectively generated accurate identifications for 20 different lipid subclasses while retaining a coverage of >98% of the lipid database entries.

## Materials and methods

### Analytical standards and reagents

Solvents and reagents used during the analysis were all MS-grade quality and are described in the Supporting Information. SPLASH LIPIDOMIX Mass Spec. Standard, a stable mixture of 14 deuterium-labeled lipids, and LightSPLASH LIPIDOMIX Quantitative Mass Spec. Primary Standard, a stable mixture of 13 endogenous lipids, were purchased from Avanti Polar Lipids, Inc. (Alabaster, AL). C17-sphinganine, palmitic acid-d31, cholesterol, and fatty acid (FA (20:4)) were purchased from Sigma-Aldrich (Steinheim, Germany). LPC(14:0), LPC(15:0), LPC(17:0), LPC(18:0), LPC(20:0), LPE(18:0), LPI(16:0), LPI(20:4), PC(16:0/16:0), PC(16:0/18:1), PC(16:0/18:2), PC(16:0/20:4), PC(16:0/22:6), PC(18:0/18:0), PC(18:0/20:4), PC(18:0/22:6), PE(16:0/18:1), PE(16:0/18:2), PE(16:0/20:4), PE(16:0/22:6), PE(18:0/18:1), PE(18:0/20:4), PE(18:0/22:6), and SM (d18:1/12:0) were purchased from Avanti Polar Lipids, Inc (Alabaster, AL). Detailed information about the chemical synthetic standards is given in supplemental Fig. S1.

#### Human plasma samples

National Institute of Standards and Technology (NIST®) Standard Reference Material (SRM®) 1950 Metabolites in Frozen Human Plasma was purchased from Sigma-Aldrich (Steinheim, Germany).

Plasma samples from 10 healthy volunteers were obtained according to Helsinki declaration and the study was approved by Aristotle University of Thessaloniki [Reference 317940/2022]. Participants were recruited at School of Physical Education & Sport Science at Thessaloniki (Greece). All participants provided informed consent.

### Lipid extraction

**Fig. 1:**
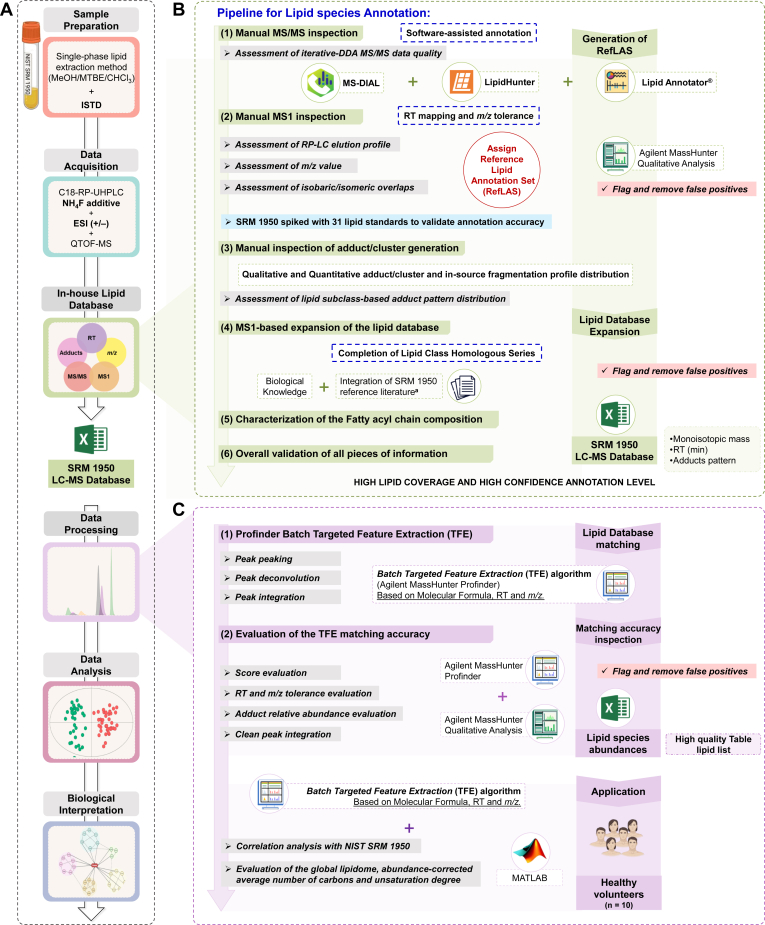
In-house SRM 1950 database-driven lipidomic workflow. A: Lipidomic workflow using a single-phase lipid extraction protocol —MeOH/MTBE/CHCl_3_, (4:3:3, v/v/v) (20)— which benefits from high coverage and simplified operations; and ammonium fluoride (NH_4_F) as mobile phases additive during sample analysis to enhance ionization efficiency (https://www.agilent.com/cs/library/applications/5991-8042EN.pdf). B: Pipeline for the generation of a high-mass resolution in-house database with high coverage and high confidence annotation level using iterative MS/MS-DDA datasets in three software annotation tools, followed by manual inspection, RT behavior, and adduct pattern. Generation of the Reference Lipid Annotation Set (RefLAS) and subsequent lipid database expansion. C: Implementation of the in-house lipid database following a matching targeted peak extraction process using the Batch *Targeted Feature extraction* (TFE) algorithm of Agilent MassHunter Profinder software based on an input table containing the 592 individual lipid species defined by their molecular formula, RT, and monoisotopic mass followed by the application on healthy volunteers. ^a^Integration of SRM 1950 published information (29, 34, 36, 37).

Lipids from SRM 1950 were extracted using a single-phase extraction protocol with a three solvent combination known as MMC method —MeOH/MTBE/CHCl_3_ (4:3:3, v/v/v)— that employs a single protein precipitation step with one-phase solvent extraction mixture (Fig. 1A) (20). Detailed information about SRM 1950 lipid extraction method, recovery determination (supplemental Table S3), and the final concentrations of the internal standards (ISTD) used (supplemental Table S4) are given in the Supporting Information.

### Sample preparation to validate annotation accuracy against lipid standards

A mixture of lipid standards containing LPC(14:0), LPC(15:0), LPC(17:0), LPC(18:0), LPC(20:0), LPE(18:0), LPI(16:0), LPI(20:4), PC(16:0/16:0), PC(16:0/18:1), PC(16:0/18:2), PC(16:0/20:4), PC(16:0/22:6), PC(18:0/18:0), PC(18:0/20:4), PC(18:0/22:6), PE(16:0/18:1), PE(16:0/18:2), PE(16:0/20:4), PE(16:0/22:6), PE(18:0/18:1), PE(18:0/20:4), PE(18:0/22:6), SM(d18:1/12:0), FA(20:4), cholesterol, and LightSPLASH LIPIDOMIX was selected for annotation validation based on exact mass and RT at MS1 scanning mode. First, a stock solution of all standards was prepared in MeOH/MTBE/CHCl_3_ (4:3:3, v/v/v). Concentrations of the lipid species were estimated based on the SPLASH LIPIDOMIX deuterium-labeled standards and its corresponding endogenous lipid species annotated in SRM 1950. From the stock solution, two serial 1:2 (v/v) dilutions were prepared. Then, three vials —containing 80 μl of the SRM 1950 lipid extract— were spiked with 20 μl of the standard solution, at high, medium, and low concentration, respectively. Final concentrations of the lipid standards in the vial were 6x(high), 3.5x(medium), and 2.25x(low) of the endogenous levels.

### Plasma fingerprinting by RP-UHPLC-ESI(+/−)-QTOF-MS analysis

Five replicates of SRM 1950 were analyzed by RP-UHPLC using an Agilent 1290 Infinity II UHPLC system coupled to an Agilent 6545 QTOF-MS (Agilent Technologies, Santa Clara) equipped with a Dual Agilent Jet Stream Electrospray source (Dual AJS ESI), (Fig. 1A). An optimized analytical method already reported in previous lipidomics studies was used (21, 22). This methodology combined acquisition in full MS-scan with automated DDA of iterative MS/MS spectra at two collision energies (20 eV and 40 eV) (21, 22).

Quality management was consistently maintained throughout the analysis process, encompassing pre-analysis, during analysis, and post-analysis phases. To ensure effective quality management, we adhered to the mQACC (Metabolomics Quality Assurance and Quality Control Consortium) guidelines (25). Detailed specifications can be found in the Supporting Information.

### Pipeline for the construction and quality assessment of the in-house lipid LC-MS database

For the development of the database, we followed a pipeline shown in Fig. 1B and comprehensively described in the Supporting Information.

Briefly, to assign lipid subclass and/or sum composition, this pipeline initially included the generation of a subset of lipid annotations —hereon referred to as Reference Lipid Annotation Set (RefLAS)— for validation of the confidence in the annotation. Overall consistency of the RefLAS was assessed by a combination of the software-assisted MS/MS spectra annotation results (supplemental Table S5) and by complementary pieces of information (*m/z* accuracy, MS/MS characteristic fragments (42), chromatographic retention behavior, and qualitative adduct and cluster profile in both positive and negative ionization modes (ESI(+/−)), always consistent with ISTD as reference compounds.

Therefore, in addition to MS/MS data, for the evaluation of LC-MS information, we used (i) RT mapping between- and within-lipid subclasses (by plotting the RT of a given lipid species against its sum composition carbon number within each unsaturation series) and (ii) *m/z* accuracy within-lipid subclass. In addition, the RefLAS was also used to characterize the qualitative and semi-quantitative adduct profiles of each lipid subclass. Theoretical *m/z* differences of all evaluated adducts and clusters, as well as the in-source fragmentation (ISF) (43, 44, 45) determined are shown in supplemental Fig. S2.

By using RefLAS as a proxy to evaluate consistency, we validated those annotations that did not accomplish the RefLAS criteria and performed a literature-based and gap-filling expansion of the database to maximize coverage, Supporting Information. To increase the level of structural information hierarchy regarding fatty acyl chain (FAC) composition, MS/MS inspection and within-sum composition RT mapping was performed (Supporting Information). To determine *sn*-position of lipids, MS/MS data was manually inspected according to already described criteria (42, 46) and the intensity ratio of fragments ions related to the positions of FAC.

### Nomenclature

For lipid category classification and nomenclature, we consistently followed the classification system established by LIPID MAPS. When describing the FAC, we also used the common names and synonyms recognized by LIPID MAPS. Specific rules for molecular species and lipid classes with known fatty acyl/alkyl constituents followed those recommended (23).

### Data pre-processing: in-house database-driven peak extraction

For data pre-processing, the in-house lipid database was used as a template to perform targeted peak extraction based on a molecular formula, mass accuracy, and RT-match approach (Fig. 1C) using the Batch *Targeted Feature Extraction* (TFE) algorithm of MassHunter Profinder software (B. 10.0.2, Agilent Technologies, Santa Clara). This algorithm employs an input molecular formula source, a .csv input file containing lipid name, molecular formula, monoisotopic mass, and RT information to extract lipids from the acquisition data files using a computational process referred to as Find Compounds by Formula.

The TFE algorithm was performed following optimized parameters detailed in the Supporting Information and briefly summarized in supplemental Figs. S3 and S4. These parameters were first optimized in both ionization modes using lipid ISTD before being applied to SRM 1950, and polarity and adduct selection was performed based on supplemental Fig. S5. Evaluation of TFE matching accuracy is described in detail in Supporting Information.

For data pre-processing evaluation, the TFE algorithm was applied to plasma samples of ten healthy volunteers to ensure and evaluate identification and match accuracy during data-preprocessing. The same parameters as those already reported for the SRM 1950 sample were followed.

Results obtained from the TFE algorithm for both the SRM 1950 sample and healthy volunteers were manually curated by performing blank subtraction and subsequent total useful signal (TUS) normalization. To evaluate the lipid profile similarity among healthy individuals and SRM 1950, we performed correlation analysis of lipid subclass total abundances as well as correlation analysis of lipid species within subclass-TUS-normalized areas. In addition, we addressed within-subclass compositional profiles (supplemental Fig. S6, Table S6) and evaluated the differences in global lipidome metrics reflecting the nature of FAC bound to lipids between lipid subclasses: abundance-corrected average carbon number and unsaturation degrees for each lipid subclass, calculated as described in (47). The Log_2_ fold change (FC) was calculated to identify the main differences among subclasses. Log_2_FC was calculated as follows: Log_2_FC = Log_2_(average lipid subclass A/average lipid subclass B) and univariate statistical analysis was performed using pairwise Mann Whitney’s U comparisons corrected by Bonferroni for multiple comparisons testing. Significance was stablished when corrected *P* < 0.05.

## Results

### In-house lipid LC-MS database and SRM 1950 lipid identification

**Fig. 2:**
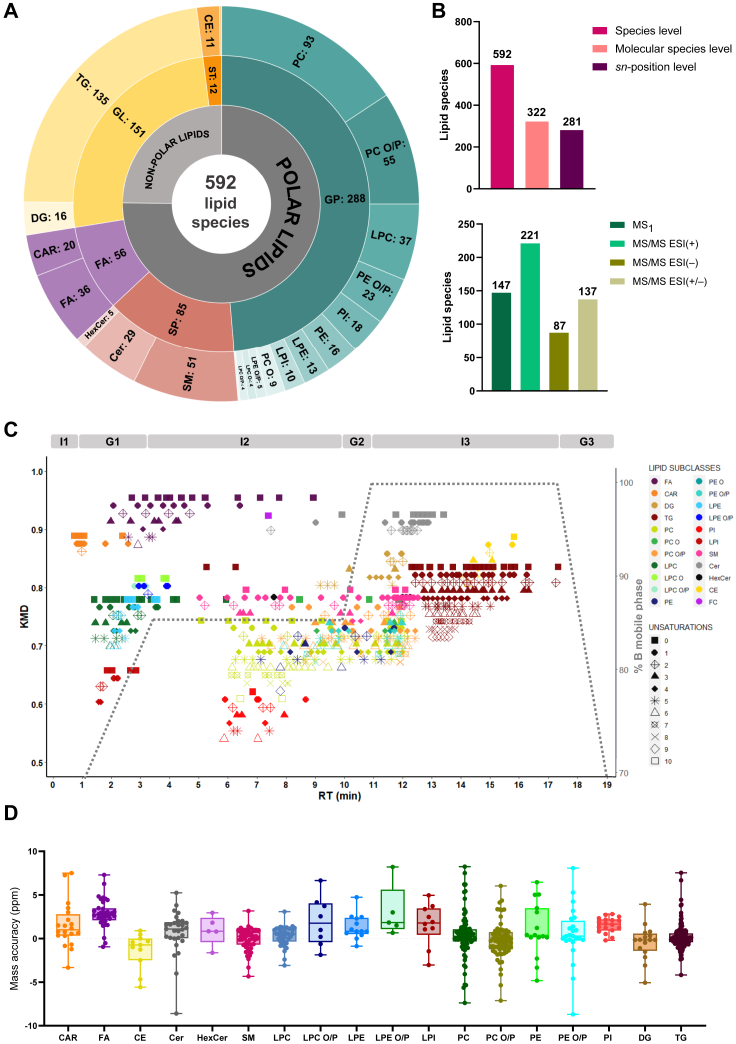
Global descriptors and indicators of the database lipid coverage, elucidation degree, and data quality. A: Number of entries subdivided by lipid type, class, and subclass. B: Hierarchy of lipid structural information –*Species level:* lowest hierarchical level. It represents the sum composition; *Molecular species level:* annotates lipid species with identified fatty acyl/alkyl residues; *sn-position level:* annotates molecular species: Fatty acyl/alkyl constituents at the glycerol backbone– and lipid species annotations associated with MS1 or MS/MS data in ESI(+) and/or ESI(−). C: Kendrick mass defect (KMD) versus RT plot showing lipid series in SRM 1950 and mobile phase composition over the chromatographic separation method: mobile phases composition: I = isocratic elution; G = gradient elution (see Supporting Information). D: *m/z* accuracy of the predominant adduct for each lipid species annotated in SRM 1950.

To achieve the highest level of data curation, we have revised our methodology and workflow, resulting in the reconstruction of our in-house lipid SRM 1950 database built using MS1 and MS/MS data obtained from RP-UHPLC-ESI(+/−)-QTOF-HRMS and encompassing 592 individual lipid molecular species (Fig. 2A, supplemental Table S2). Within the whole lipid dataset, 289 annotations fulfilled complete agreement and consistency in the four pieces of information evaluated: mass accuracy, MS/MS characteristic fragments, chromatographic retention behavior, and qualitative adduct and cluster profile in both the positive and negative ion modes (ESI(+/−)), therefore constituting the RefLAS (supplemental Table S7).

In addition, some of the in-house database annotations were validated against 31 lipid standards from 10 lipid subclasses in both ionization modes (when available for the lipid subclass). Thus, validation was performed by spiking SRM 1950, using MS1 scanning mode and matching exact mass and RT.

The total of 592 lipid annotations present in our database encompassed five of the eight lipid categories, (Fig. 2A, supplemental Table S2), containing a total of 20 lipid subclasses: in the category of fatty acyls, we report fatty acids (FA, 36 species) and carnitines (CAR, 20 species); in the category of sphingolipids (SP), our database is composed of ceramides (Cer, 29 species), sphingomyelins (SM, 51 species), and hexosylceramides (HexCer, 5 species). Within the category of glycerophospholipids (GP), we report diacylglycerophosphocholines (PC, 93 species), acyl/alkyl/alkenylglycerophosphocholines (PC O/P, 64 species), diacylglycerophosphoethanolamines (PE, 16 species), acyl/alkyl/alkenyl-glycerophosphoethanolamines (PE O/P, 24 species), diacylglycerophosphoinositols (PI, 18 species), and their corresponding monoacyl/alkyl/alkenylglycerophospholipids as LPC (37 species), LPC O (4 species), LPC O/P (4 species), LPE (13 species), LPE O/P (5 species), and LPI (10 species); the category of glycerolipids (GL) was represented by di- and tri-glycerides (DG and TG, 16 and 135 species, respectively); we only report cholesteryl esters (CE, 11 species) and cholesterol as representatives of the sterols (ST) lipid class. In accordance with previous studies (29, 34, 36, 37), TG and PC were the most represented subclasses in our database (22.80% and 15.71% of lipid species, respectively), followed by PC O/P (10.81% of the total number of lipids identified).

The lipids present in our database were annotated with different levels of the lipid structural information hierarchy (https://lipidmaps.org/lipid_nomenclature/rules/hierarchy) (23), as 322 lipids (54.39%) were annotated up to the molecular species level (species identified with fatty acyl/alkyl residues) and 281 (47.47%) at a structural level equivalent to the *sn*-position level (fatty acyl/alkyl constituents at the glycerol backbone or localization of sphingoid base backbone and fatty acyl chain in SL) (Fig. 2B).

### Quality assessment of the lipid coverage within the lipidome chemical space: evaluation of the extraction method for the recovery of lipids from SRM 1950

To ensure a high chemical space coverage of SRM 1950 lipidome, our workflow started with the selection of a single-phase lipid extraction method —such as the MeOH/MTBE/CHCl_3_ (MMC) solvent system (20)— and an optimized analytical methodology that included ammonium fluoride (NH_4_F) as mobile phase additive to enhance ionization efficiency (https://www.agilent.com/cs/library/applications/5991-8042EN.pdf). This was proven with the SRM 1950 lipid recovery results, using the SPLASH LIPIDOMIX mixture as ISTD. The MMC-based lipid extraction method provided recovery rates of 90.7%–103.9%, demonstrating a good coverage of the polarity range across all detected lipid species (supplemental Fig. S7, Table S3). PS, PG, MG, and PA were not detected during data processing; this could be due to the ionization capacities of these lipids, not enough to be detected at the relatively low physiological concentrations present in human plasma (supplemental Table S3) considering an untargeted methodology. Also, CE and cholesterol were not reported due to its high degree of ISF.

### Quality assessment of orthogonal properties for annotation of the lipid subclass and sum composition

#### Assessment of MS/MS data quality

Prior to manual curation of data, automated lipid identification software —Lipid Annotator, MS-DIAL, and LipidHunter— provided a total of 445 annotations supported by iterative exclusion DDA dataset. These annotations were subjected to curation criteria by checking their conformity with the characteristic fragmentation patterns described in the literature and in www.lipidomicstandards.org (48, 19). These criteria were fully achieved by our initial RefLAS of lipid annotations (supplemental Table S2). All 289 lipid annotations from RefLAS and 75.17% of the total annotations present in our in-house database had an associated MS/MS spectrum in at least one polarity (Fig. 2B, supplemental Table S7), thus proving a high richness of MS/MS data associated to the lipid annotation dataset when compared to other SRM 1950 characterizations (29, 34, 36, 37).

#### Assessment of isobaric and isomeric overlaps of lipids during manual MS data inspection

During manual MS1 and MS/MS inspection, several interferences were detected, highlighting mainly isobaric and isomeric overlaps, leading to a more complicated data interpretation and making lipid characterization a challenging task (49), supplemental Fig. S8. Misidentifications have been discussed in several studies and represent an ongoing issue (17, 49, 50), an example of misidentification is given in supplemental Fig. S9. Therefore, in parallel to the construction of the database, an in-depth manual examination of the MS1 and MS/MS data was performed for the systematization of the downstream data curation pipeline, including the resolution of all detected types of isobaric/isomeric overlaps (supplemental Fig. S10) and the recognition of ISF (supplemental Fig. S11) (45). On the one hand, isobaric lipids —which do not have exactly the same elemental composition— can be solved with adequate MS1 data handling and sufficiently high mass resolution, while on the other hand, resolution of isomeric species —which do have exactly the same elemental composition— relies always on the use of MS/MS spectra in both conventional and advanced MS strategies.

Selected examples to illustrate this section during MS manual curation are shown in detail in supplemental Fig. S12-16.

#### Assessment of reversed-phase chromatographic separation: elution profile of lipids

In addition to MS/MS data, RT in RPLC is an orthogonal piece of information that is well documented to improve the confidence in the annotation within lipidomic studies (51). Noteworthily, our chromatographic gradient is optimized to cover a wide range of analyte polarities in a short method runtime of 19 min, while favoring the separation of mid-polarity lipids (diacyl-GP, SP) and low-polarity TG, that constitute the majority of lipid species detected in untargeted lipidomic studies of human samples. For this purpose, instead of a continuous gradient separation, the chromatographic method is segmented in six well-differentiated time segments which influence the overall chromatographic behavior of lipid species: a combination of three isocratic elution segments and three gradient elution segments (Fig. 2C and supplemental Fig. S17, I1 [0–1 min], G1 [1–3.50 min], I2 [3.5–10 min], G2 [10–11 min], I3 [11–17.10 min], and G3 [17.10–19 min], by their order according to the RT dimension). After having performed MS/MS validation of our preliminary RefLAS, we further demonstrated its consistency in RT behavior according to the lipid subclass, as the elution of compounds within distinct lipid subclasses was in consistency with what has been extensively described in the literature for RPLC-MS lipidomics following this method (22, 52). These lipid subclass elution profiles were also consistent with predicted Chemicalize logD values (https://chemicalize.com/) (Table 2, supplemental Fig. S18). RefLAS lipid series further validated by RT mapping were used to evaluate within-series consistency of non-RefLAS annotations.

**Table 2:** Ranges of RT and ECN within each lipid subclass detected in SRM 1950

Abbreviation Subclass	n^c^	Gradient Region of Elution^d^	RT Range			ECN Range			logD^e^
			RT_min_	RT_max_	Δ^RT^	ECN_min_	ECN_max_	Δ^ECN^	
LPI	10	G1	1.57	2.84	1.27	12	18	6	−0.99
CAR	20	I1-G1	0.73	2.58	1.85	2	16	14	2.78
LPC	37	G1-I2	1.42	5.94	4.52	10	24	14	3.22
LPE	13	G1-I2	1.98	2.55	0.57	10	18	8	3.53
LPC O/P^c^	8	G1-I2	2.80	3.88	1.08	14	18	4	3.59
LPE O/P^c^	5	G2	2.95	3.93	0.98	14	16	2	3.91
FA	36	I1-G1-I2	1.97	8.94	6.97	10	26	16	5.60
PI	18	I2	5.99	8.97	2.98	26	34	8	6.62
Cholesterol	1	I2	7.40	7.40	0.00	25	25	0	7.11
HexCer	5	I2-G2-I3	7.44	12.22	4.78	32	38	6	9.09
SM	51	G1-I2-G2-I3	5.03	12.54	7.51	28	42	14	9.20
PC	93	I2-G2-I3	5.08	12.05	6.97	22	36	14	10.14
PE	16	I2-G2	7.14	11.70	4.56	22	34	12	10.45
PC O/P	69	I2-G2-I3	7.17	12.44	5.27	19	39	20	10.53
PE O/P	24	I2-G2	7.82	11.97	4.15	24	32	8	10.83
Cer	29	I2-G2-I3	7.60	13.47	5.87	30	46	16	11.07
DG	16	I2-G2-I3	9.17	12.00	2.83	26	32	6	12.00
CE	11	I3	14.09	15.84	1.75	10	16	6	14.03
TG^a^	2	I2	5.27	6.27	1.00	24	26	2	-
TG^b^	133	I3-G3	12.32	17.34	5.02	36	54	18	18.92

a TG(8:0/8:0/8:0) and TG(8:0_8:0_10:0).

b Rest of TG lipid species.

c n = number of lipid species identified in each subclass.

d RT of gradient region of elution: I = Isocratic; G = Gradient. I1 = [0–1 min]; G1 = [1-3.5 min]; I2 = [3.5-10 min]; G2 = [10–11 min]; I3 = [11-17.10 min]; G3 = [17.10-19 min].

e logD values were predicted using Chemicalize (https://chemicalize.com). Results refer to example lipids bearing C16:0 fatty acyl chain (e.g. PC(16:0/16:0), CE(16:0), TG(16:0/16:0/16:0), SM(d18:0/16:0), etc…).

After lipid database completion, we characterized the whole elution profile of lipid subclasses comprehensively (Fig. 2C and supplemental Fig. S17), observing that most lipid subclasses eluted in more than one elution region (Table 2 and supplemental Fig. S17) despite the partial overlap between lipid subclasses. Lower RT were observed for the monoacyl lipid species —most polar lipid subclasses with relatively low equivalent carbon number (ECN, equal to the sum composition carbon number – 2·number of unsaturations) (lysoGP, CAR, FA)— eluting at the beginning of the chromatogram, followed by diacyl lipid species (mid ECN) and SP eluting in a wide RT range mostly corresponding to the isocratic I2 elution region, and then followed by triacyl lipid species (relatively high ECN), mostly eluting in the isocratic I3 elution region. However, an exception was CE, eluting at the isocratic I3 elution region while showing lower ECN than TG, as the ECN of CE does not contemplate the hydrophobic nature of the cholesterol moiety found in CE. Short RT ranges for the lipid subclass correlated with (i) relatively low number of lipid species within the subclass, (ii) preferential elution in a non-isocratic region and (iii) poor retention (in the case of CAR). Conversely, highly represented subclasses exhibited RT elution ranges > 6 min, such as in FA, PC and SM (supplemental Table S2), suggesting that underrepresented lipid classes may in fact have higher retention time ranges, but most species fall below the limit of detection. As an illustrative example, we determined the presence of two TG with low sum composition carbon number at earlier retention times from the main TG elution region (Fig. 2C and supplemental Fig. S17). This reinforces the idea that annotations outside the gross RT range of the lipid subclass should not be systematically addressed as false positives if justified by the predicted polarity, especially when performing database expansion toward lipid species containing short- and very long-chain fatty acids.

Aside from the differences in RT attributable to the functional groups of each lipid subclass, we evaluated the effect of the sum composition within each unsaturation series of each lipid subclass using RT mapping (supplemental Fig. S19). The effect of higher sum composition carbon number —higher retention— and higher unsaturation degree —lower retention— is well documented in RPLC-MS (37). We used the sum composition-based elution order in the RT dimension independently of the chromatographic gradient region to reject false positives and provide consistency in the assignation of RefLAS for each lipid subclass. In addition, RT mapping was validated by agreement with the elution order of ISTD within the same lipid subclass (supplemental Fig. S19).

By using the RT mapping of RefLAS (289 entries) and ISTD (14 deuterated compounds) as a surrogate, we were able to assess the quality of non-RefLAS lipid annotations provided by software and annotations obtained during literature-based and subsequent gap filling-based database expansion to 592 lipids (Figs. 1 and 2). Consequently, all the lipid species included in our database were consistent in terms of retention behavior of the sum composition within each lipid subclass (supplemental Fig. S19).

#### Assessment of MS1 m/z value: error consistency within lipid subclasses

Mass accuracy of the MS1 lipid-related adduct ions was considered reliable when the error fell within the range of ±10 ppm, (Fig. 2D). Inspection of the relative *m/z* error of the predominant adduct ions (supplemental Table S7) revealed differences between lipid subclass in terms of average and dispersion values, where most annotations fell within a range lower than ± 5 ppm from the lipid subclass error mean (Fig. 2D). We found high consistency in the *m/z* errors of predominant adduct ions attributable to each lipid subclass, as 96.28% (570 out of 592) of the lipid species had Z-score values < 2. From the 22 lipid species outside Z-score > 2, only 9 species showed Z-score values > 3 (supplemental Table S7). Thus, by contextualizing the *m/z* error of annotations within their corresponding subclass, we demonstrated high consistency in our database.

#### Assessment of generation of adducts, clusters and in source-fragmentations: Lipid subclasses-based pattern distribution

During LC-ESI-MS analyses, protonation and deprotonation processes are not the only ionization events occurring in the ESI source. Background ions such as metals, impurities, and co-eluted species can react with analytes, leading to the formation of adducts, oligomers, and clusters causing data redundancy (supplemental Fig. S8). Nonetheless, redundancy from these rich ESI-source adduct and cluster profiles can be exploited for lipid subclass assignment, as both the chemical structure of specific lipid subclasses and the applied LC-MS method are known to pose intrinsic and method-dependent constraints in adduct/cluster formation (21, 53, 54).

The lipidomics community has exploited for data curation the general qualitative constraints that confer distinctive adduct patterns to lipid subclasses analyzed under a specific LC-MS method (21, 48). Previous attempts to systematize this information in a quantitative and method-dependent manner (26), have in fact stated limitations associated to the reproducibility of adduct intensity order and the different orders of magnitude at which such adducts appear in MS data (27).

**Fig. 3:**
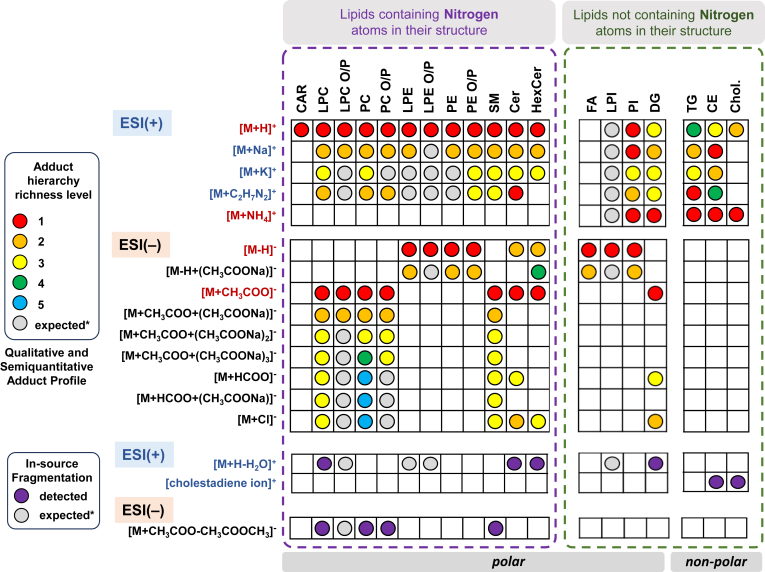
Adduct profile hierarchy and in-source fragmentation (ISF) presence in ESI(+/−) in all lipid subclasses present in our LC-MS human plasma lipid database. ∗Expected adducts but not detected during manual inspection. n = 3–6. Sodium acetate clusters (+82.0031 Da), observed in LC-MS background, appear as repeating units (n = 1–6) in ESI(−). Adducts marked in *red* correspond to those of hierarchy level 1 in both polarities.

As a novel approach ensuring the highest degree of data consistency and minimal false positive annotations, we aimed to systematize the quality assurance based on adduct information in a method-dependent, two-step data curation strategy: in our first adduct profile validation step, we generated consensus qualitative adduct formulas (cQualForm) associated to each lipid subclass (Fig. 3, supplemental Table S8), considering for their generation RefLAS annotations (supplemental Table S8). Only annotations where molecular mass was unambiguously determined by the co-presence of two or more adducts were included in the RefLAS. As expected, each cQualForm was associated with the capability for adduct formation in each lipid subclass, which is determined by those functional groups outside the FAC (e.g., the choline head of PC). An exception is those ester and ether groups inherent to FAC (e.g., in TG) (Fig. 3). Since the most abundant lipid molecular species for each subclass were included in the RefLAS, the presence of adducts absent in the associated cQualForm were used for false-positive identification in annotations not included in the RefLAS. Secondly, within each RefLAS lipid subclass annotation set, we evaluated the order of adduct intensity to generate consensus semi-quantative adduct formulas (cSquantForm) representing reproducible adduct hierarchy (supplemental Table S8) in all the entries belonging to each reference dataset and are used to categorize the adduct hierarchy richness level (AHRL) according to the levels at which one or more adducts appear in the formula (Fig. 3).

Surprisingly, cSquantForm at maximum abundances were mostly lipid subclass-specific, except between lysoGP and their diacyl equivalents as well as between alkyl/alkenyl ether lipids and their acyl counterparts (Fig. 3). By their comprehensive evaluation, we determined differences in the adduct pattern between SM and the group of lipid subclasses conformed by PC, PC O/P, LPC, and LPC O/P, undistinguishable by their qualitative adduct profile, as SM showed a unfavored formation of [M+C_2_H_7_N_2_]^+^ compared to other adducts (Fig. 3). This effect of unfavored [M+C_2_H_7_N_2_]^+^ was additionally observed between TG and CE (Fig. 3). In addition, adduct consistency for low-abundance annotations was accompanied by the co-occurrent AHRL values observed (supplemental Table S8). cSquantForm compliance between RefLAS and non-RefLAS annotations was observed for 97.44% of our lipid database entries, with the exception of 10 TG (7.41% of TG annotations) —which showed [M+K]^+^ peak intensities higher or equal to [M+Na]^+^ but maintained overall higher-order adduct hierarchy— and 3 CE (27.27% of CE annotations), showing unusually high [M+K]^+^ intensities.

In addition to the cQualForm, cSquantForm and AHRL, we introduced an additional metric for MS1 assessment of lipid subclass by evaluating the qualitative profile of ISF indicative of lipid subclass according to the Lipid Standard Initiative (LSI) (19). From these ISF, within our method, we observed [M+H-H_2_O]^+^ ions from lipids containing a hydroxyl (–OH) group: LPC, Cer, HexCer, DG, and cholesterol. Moreover, the ISF of acetate adducts of choline-head containing lipids —SM, PC, PC O/P, and LPC— results in the loss of the anion (CH_3_COO^-^) and elimination of a methyl group from the quaternary nitrogen of the choline head group [M-15]^-^.

Overall, ISF information was combined with adduct and cluster profile data in order to provide an additional MS1-based layer of confidence and consistency in the annotation. We have proved that adduct and cluster hierarchy generates reproducible patterns within our method (Fig. 3), demonstrating that the use of internal standards and RefLAS compliance to define and evaluate adduct profile and hierarchy is a viable and complementary strategy to assess lipid annotation confidence, within the applied method.

#### Assessment of the impact of the fatty acyl chain composition

Once the belonging of specific lipid subclasses and sum composition was assessed, the comprehensive assessment of annotation levels was performed. Specific fragment ions derived from the loss of fatty acyl substituents as well as their intensity fragment ratios (55), were evaluated in MS/MS spectra from ESI(+/−). In addition, individual RT mapping of each lipid subclass was performed to address the elution order based on the number of carbons and unsaturation within each subclass (supplemental Fig. S19).

**Fig. 4:**
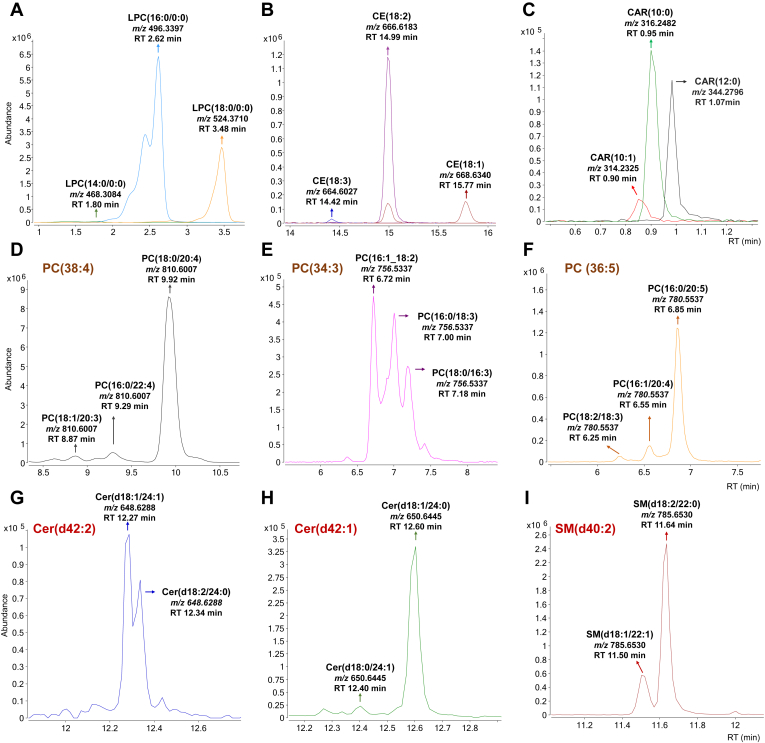
RT elution pattern detected during manual inspection. A: Elution pattern for lipid subclasses containing one fatty acyl chain at the same degree of unsaturation. B: Elution pattern for lipid subclasses containing one fatty acyl chain at the same number of carbons. C: Example of elution pattern in carnitines when having different degree of unsaturation and number of carbons. D-F: Elution pattern of isomers of glycerophospholipid subclasses containing two fatty acyl chains having same chain length but different unsaturation degree on them and same unsaturation degree but different carbon number. The elution pattern is shown in (D) PC(38:4), (E) PC(34:3), (F) PC(36:5). G-I: Elution pattern of isomers of sphingolipid subclasses having same chain length but different unsaturation degree on them. The elution pattern is shown in (G) Cer(d42:2), (H) Cer(d42:1), (I) SM(d40:2).

To confirm annotation of those lipid species containing one FAC (LysoGP, CAR and CE), RT elution pattern is well known since at the same degree of unsaturation, a higher number of carbons leads to a higher retention in RPLC, as shown in Fig. 4A (e.g., decreasing elution order LPC(18:0/0:0) (RT 3.48 min) > LPC(16:0/0:0) (RT 2.62 min) > LPC(14:0/0:0) (RT 1.80 min)). In addition, within lipids with the same number of carbons, a higher unsaturation degree leads to a lower retention (Fig. 4B (e.g., decreasing elution order CE(18:1) (RT 15.77 min) > CE(18:2) (RT 14.99 min) > CE(18:3) (RT 14.42 min)). An additional example containing both different unsaturation degree and number of carbons is shown with carnitines in Fig. 4C (e.g., CAR(12:0) (RT 1.07 min) > CAR(10:0) (RT 0.95 min) > CAR(10:1) (RT 0.90 min)).

For those GP subclasses with two FAC, multiple structural isomers were annotated as having identical sum composition but different FAC length, *sn*-position and/or unsaturation degree. This allowed us to perform a comprehensive inspection of the retention behavior between lipid chain isomers within the PC subclass that could be extrapolated to the rest of GP subclasses containing two FAC. For isomers having the same chain length but different unsaturation degrees, we observed higher retention times when the FAC had higher differences in the unsaturation degree between them (e.g., (18:2_16:0) > (18:1_16:1)). Additionally, for isomers bearing the same unsaturation degree on their FAC, we observed higher RT for isomers containing higher carbon number in the FAC with less unsaturation degree (e.g., (18:1_16:1) versus (20:1_14:1)). Examples of RT behavior within lipid isomers of the same sum composition are shown in Figure 4D-F.

On the other hand, SP subclasses were observed to behave opposite to GP. This reversal occurs because the sphingoid base backbone possesses a double bond in *trans*-configuration at C4, whereas the double bond of GP, in general, exists as *cis*-configuration isomers (56). For those SP containing the same FAC length but different unsaturation degrees on the FAC, a higher RT was observed when there was a higher unsaturation degree on the acyl chain of the backbone as shown in Figure 4G-I. By following this expected elution pattern in diacyl lipid species, accuracy of FAC annotation between lipid isomers having identical sum composition was confirmed and our database was curated accordingly.

Finally, another major challenge in conventional lipidomics studies lies in the annotation of TG species and their multiple regioisomers resulting from the combination of different FAC there is already significant variability in the way this lipid subclass is reported in previous studies. Unfortunately, serious limitations are present in the approaches reported which largely depend on chromatographic separations (57).

**Fig. 5:**
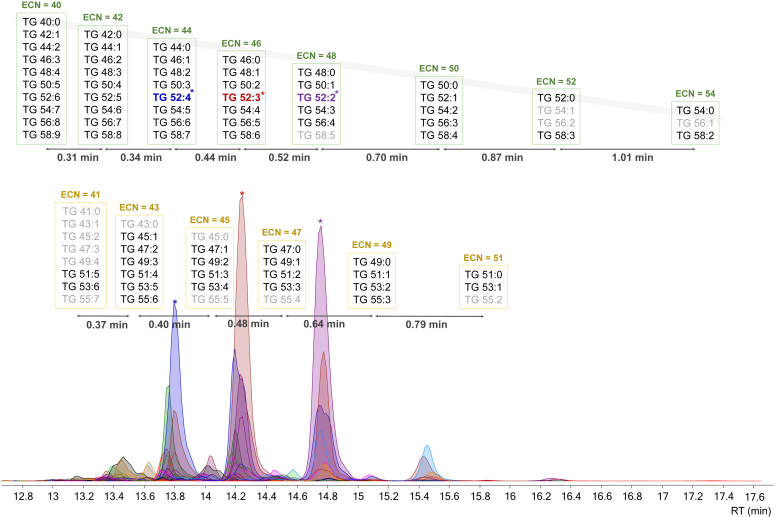
TG co-elution due to ECN-series separated in even and odd carbon numbers. TG marked in *light gray* are not detected in this analysis but are expected to co-elute within its ECN-serie. The general expression of ECN is ECN = NC − 2 × DB where NC and DB are the total number of carbons and the number of double bonds, respectively. ∗More abundant TG highlighted in bold font.

During SRM 1950 analysis we have observed co-elution at the same RT (± 0.2 min) of TG with the same ECN (e.g., TG(48:0), TG(50:1), TG(52:2), TG(54:3) and TG(58:5) all with an ECN = 48) as shown in Figure 5, allowing us to establish ECN-based series. In addition, within these series, as the ECN increases, the difference in RT between the series also increases; for example, a higher difference in retention is observed between 54- and 52-ECN series than between 40- and 42-ECN series. Furthermore, within species with the same sum composition carbon number, it can be observed that a higher degree of unsaturation results in lower retention (e.g., TG(52:2) (RT 14.74 min) > TG(52:3) (RT 14.24 min) > TG(52:4) (RT 13.79 min)) as already reported with monoacyl- and diacyl-chain lipid subclasses.

**Fig. 6:**
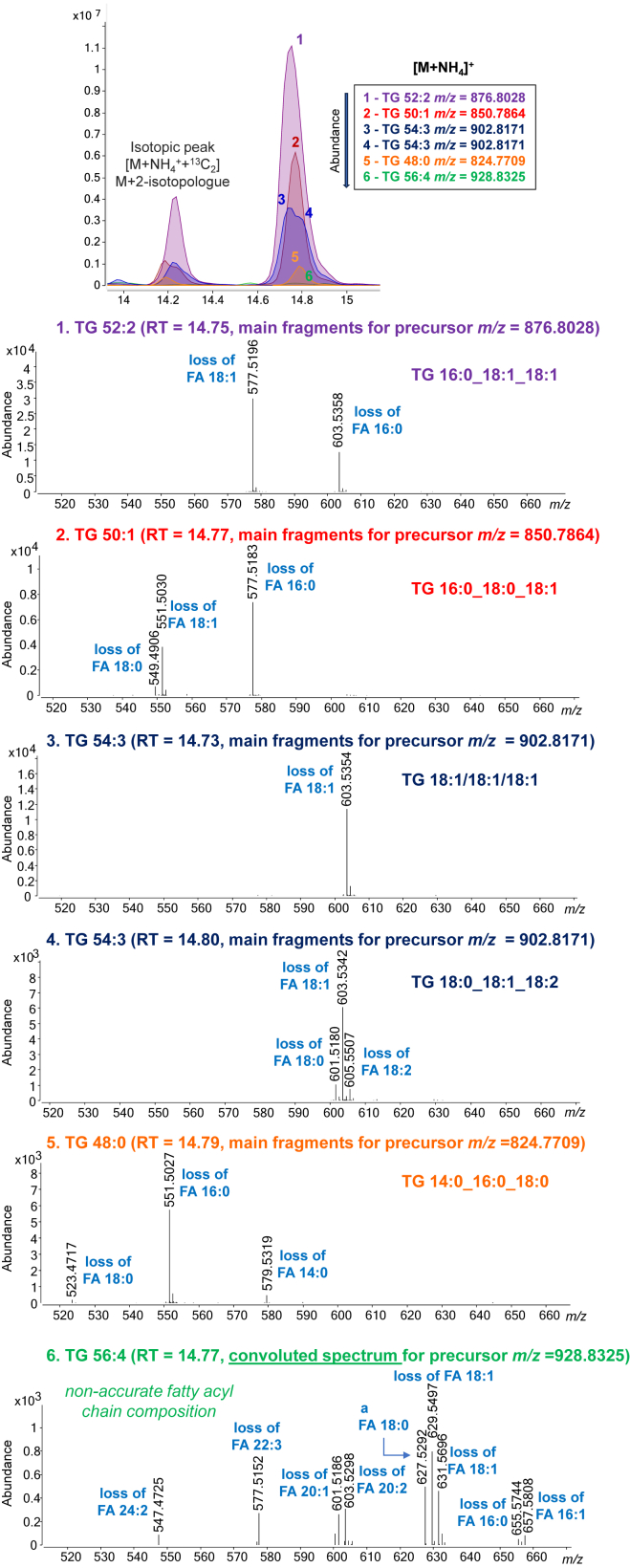
Example TG co-elution of ECN-series leading to clean and MS/MS convoluted spectra.

Adding to this complexity, the presence of isotopic overlaps with various TG further complicates the analysis, ultimately resulting in the contamination of MS/MS data by all co-eluting TG species (58, 59). This leads to challenges in characterizing the FAC present in low-abundance TG. While TG annotation is based on the neutral losses of free fatty acids from the precursor ammonium adduct [M+NH_4_]^+^, it has been observed that in most cases the fragments observed in MS/MS spectra are always the same but depending on the precursor ion that is related with, it corresponds to a certain FAC (e.g., fragment *m/z* 577.5196 when related to [M+NH_4_]^+^ TG(52:2) (*m/z* 876.8028) corresponds to NL of FA(18:1); while when related to [M+NH_4_]^+^ TG(50:1) (*m/z* 850.7864) corresponds to NL of FA(16:0)). This interference, together with the co-elution of the ECN series, can lead to extremely complicated chimeric spectra containing even 8 fragment ions that correspond to different NL of FA resulting in the misinterpretation of MS/MS spectra. An example of TG co-elution of ECN series leading to clean or convoluted MS/MS spectra is shown in Figure 6, where the most abundant TG have clean spectra while the less abundant ones show fragment contamination. Regarding all this information, we suggest reporting TG as sum composition unless non-convoluted MS/MS spectra is obtained that allows the unambiguous characterization of the FAC composition.

### Data pre-processing: in-house lipid LC-MS database-driven peak extraction

Even though different approaches to develop lipid databases to facilitate the annotation process have been already reported, less information is available regarding their use during data pre-processing as a strategy to increase data quality prior to statistical analysis.

The development of a database based on molecular formula, monoisotopic mass, and RT, rather than conventional MS/MS spectra or fragment-based libraries, has provided us with a distinctive advantage during data pre-processing, by focusing on a molecular formula and RT match-based approach using the TFE algorithm of Agilent MassHunter Profinder software (supplemental Fig. S3). First, to determine and evaluate the lipid species abundances of the lipid profile in SRM 1950, our lipid database was used for the targeted reprocessing of data from five SRM 1950 technical replicates. Average blank samples were subtracted from each replicate and the peak area of individual lipid species was assessed and was represented in supplemental Fig. S6. To assess the presence of oxylipins in SRM 1950 plasma sample, a targeted analysis was conducted using a database of 69 oxylipin standards as a template (supplemental Table S9) and the TFE algorithm, applied to the five SRM 1950 technical replicates in ESI(−). As a result, no oxylipins were detected, likely due to their very low physiological concentrations or being below the limit of detection (LOD). In addition, assessment of RT variability for each lipid specie in the database was calculated considering the five technical replicates of SRM 1950. The average RT uncertainty according to the confidence interval (CI_95%_) was 0.046 min (CI_95%, max_ – CI_95%, min_), with a standard deviation of 0.033 min. None of the lipids surpassed a CI_95%_ of 0.2 min, thus proving the robustness of the method in terms of RT (supplemental Fig. S20).

To validate the performance of our LC-MS database, plasma samples from ten healthy volunteers were analyzed, and data was processed using the TFE algorithm. Notably, 583 out of the 592 lipid species were detected in healthy individuals, which demonstrated a remarkable coverage reproducibility (>98%). Such coverage illustrated the reliability and effectiveness of the database in accurately locating chromatographic peaks associated to lipid molecular species annotated during SRM 1950 characterization, with overall minimal curation efforts. Thus, we found compelling evidence that combining our lipid database with the TFE algorithm renders a fast and robust data processing method, which can be fully integrated in our lipidomic workflow, Figure 1.

**Fig. 7:**
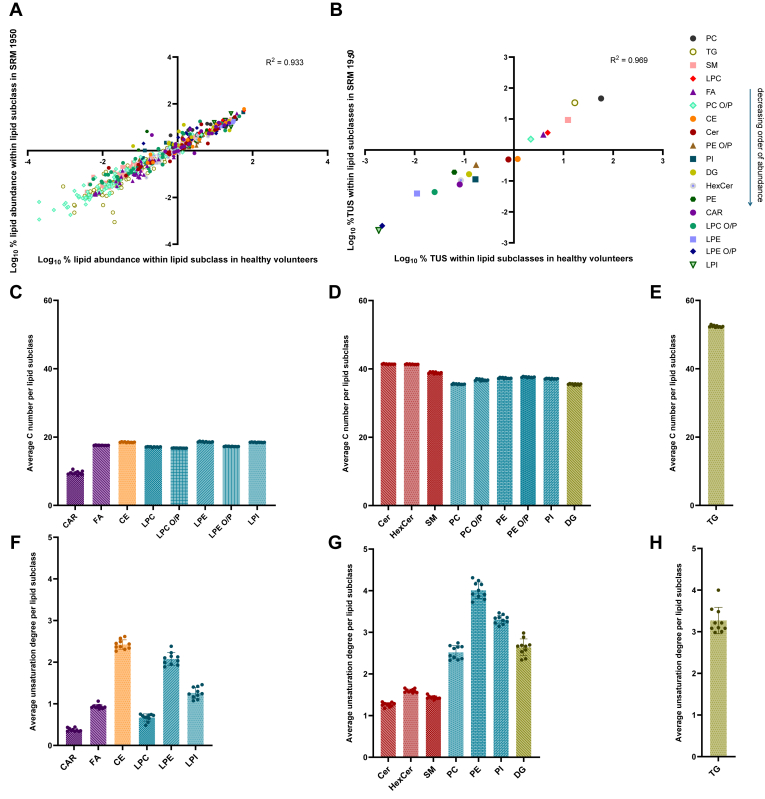
Healthy volunteer’s lipid profile evaluation. A: Correlation of individual lipid species normalized log abundance percentage between healthy volunteers and SRM 1950. B: Correlation of lipid subclasses normalized TUS log-abundance percentage between healthy volunteers and SRM 1950. C: Abundance-corrected average carbon number of monoacyl lipid subclasses in healthy volunteers. D: Abundance-corrected average carbon number of diacyl lipid subclasses in healthy volunteers. E: Abundance-corrected average carbon number of triacyl lipid subclasses in healthy volunteers. F: Abundance-corrected average unsaturation degree of monoacyl lipid subclasses in healthy volunteers. G: Abundance-corrected average unsaturation degree of diacyl lipid subclasses in healthy volunteers. H: Abundance-corrected average unsaturation degree of triacyl lipid subclasses in healthy volunteers. ∗In (F) and (G), LPC O/P, LPE O/P, PC O/P, and PE O/P were not plotted since fully identification was not achieved.

To assess the results obtained from the 10 healthy individuals and determine their similarity with SRM 1950, data from TFE was curated (Supporting Information), following blank subtraction and total or within-subclass TUS normalization (Fig. 7A, B, respectively). We found high correlation values between the relative abundances of each lipid subclass in SRM 1950 and healthy volunteers (Fig. 7A), demonstrating our method determined a similar composition (*R*^2^ = 0.97) in terms of lipid subclass relative distribution (supplemental Table S10). In addition, a high correlation value (*R*^2^ = 0.93) between the relative abundances of individual lipid species within each lipid subclass, proving that their relative distribution is similar between SRM 1950 and healthy volunteer plasma lipid extracts. Collectively, these correlations corroborate that similar lipidome profiles can be obtained when analyzing individual plasma samples from healthy donors when compared to RM, supporting a direct applicability of the method for real clinical samples in comparative studies.

To illustrate the capabilities of the combination of our method and database, we aimed to provide general descriptors of the lipidome of healthy volunteers where contribution of individual lipid species to the general properties was required (Fig. 7C–H). This allowed us to provide an estimation of the average number of carbon atoms present in fatty acyl chain residues for the measured subclasses with very low dispersion among healthy individuals, (Fig. 7C–E, supplemental Table S11 and S12). Within monoacyl/alkyl/alkenyl lipids (Fig. 7C), CAR displayed the lowest average carbon number (C = 9.50, Log_2_FC < 0.8 when compared to all monoacyl/alkyl/alkenyl lipids), indicating, as expected, shortage of FAC due to beta-oxidation. The rest of monoacyl/alkyl/alkenyl lipids showed an average carbon number (17.63 ± 0.74), where slightly longer FAC were found to be bound to CE, LPE, and LPI with respect to LPC, LPC O/P and LPE O/P (Fig. 7C); however, statistical significance (*P-*value < 0.05) was found in all comparisons except when comparing LPI and LPE (supplemental Tables S10). In the case of diacyl/alkyl/alkenyl lipid subclasses (Fig. 7D) the average carbon number was 37.9 ± 2.2, from which the SP category showed slightly higher carbon number compared to GP and glycerolipid subclasses (40.5 ± 1.5, *vs* 36.9 ± 0.8 and 35.5 respectively). Results of the statistical analysis indicated significant differences in most of the comparisons except for PI versus PE and PC O/P, Cer versus HexCer, DG versus PC and PE versus PE O/P that resulted in *P*-values > 0.05 indicating similarities among their abundance-corrected average number of carbons (supplemental Table S10). Regarding TG (Fig. 7E), the only triacyl lipid subclass identified, an average carbon number of 52 was observed.

With respect to the unsaturation degree, more differences were observed in the results. In monoacyl lipids (Fig. 7F) CE and LPE resulted to be the lipid subclasses with higher average unsaturation degree (2.42 and 2.08 respectively), both having a Log_2_FC > 2 when compared to CAR that resulted the monoacyl subclass with the lower unsaturation degree (0.94). In addition, all comparisons resulted significant after statistical analysis (supplemental Table S10). In the case of diacyl lipid species (Fig. 7G), even though a significant difference (*P*-value < 0.05) was only observed between DG and PC (supplemental Table S10), a clear distinction was observed between different lipid categories, with a lower unsaturation degree in SP subclasses (1.43 ± 0.17) —SM, Cer and HexCer—, and a higher unsaturation degree in the rest of diacyl lipid subclasses (3.12 ± 0.69). Within all of them, PE followed by PI resulted to be the diacyl subclasses with a higher unsaturation degree (4.01 and 3.31 respectively) observing in the three cases a Log_2_FC > 1 when compared to all SP subclasses detected. Finally, in the case of TG (Fig. 7H) an average unsaturation degree of 3.27 was detected.

Altogether, these results manifest that the use of our database is able to provide both highly-curated, global (Fig. 7C-E, supplemental Tables S11 and S12) and individual lipid species abundance-corrected metrics (Supplemental Tables S11 and S12) reflecting lipid-bound carbon pools which may be mobilized among different subclasses, as well as their differences in unsaturation degree, which have been described to be altered in diverse clinical conditions (60, 61).

## Discussion

A deep profiling of the SRM 1950-specific lipidome is essential not only for supporting our understanding of normal lipid metabolism but also for advancing our knowledge of lipidome-related changes in upcoming clinical investigations. Therefore, to assess the validity and capability of our in-house database, the list of identified lipid species considering lipid extraction, analytical method, and annotation strategy was compared with results from previous literature (supplemental Table S1) (29, 34, 36, 37).

In comparison with previous characterizations of SRM 1950, our in-house database provides the highest coverage up to date within libraries with high degree of curation (29, 34, 36, 37) and proves representation of major lipid subclasses found in other studies (supplemental Fig. S21, Table S1). Furthermore, we determined a higher number of lipid species in a shorter total method runtime (except compared to the method reported by Chen *et al.*, (29)). The absence of oxylipin detection is consistent with previous studies on the SRM 1950 lipidome, where no oxylipins were identified. This suggests that oxylipin levels in healthy individuals remains extremely low and, therefore, the use of instruments such as LC-MS/MS with a triple quadrupole could be recommended to improve detection sensitivity and specificity (62).

In terms of lipid extraction methods, we followed a simplified single-phase extraction procedure with no re-dissolution step that offers comparable lipidome coverages to biphasic extraction methods while overcoming their intrinsic disadvantages (29, 36). Notably, only one analytical platform was used for lipidome profiling when compared to previously reported analyses (34), and no ion mobility was used to help in the resolution of isomeric interferences (29), that were indeed assessed manually. Moreover, lipid annotation confidence surpassed previously reported identifications, as our in-house database incorporated the highest number of complementary pieces of information for a LC-ESI-HRMS platform, including information highly influenced by the method (RT and adduct profile and hierarchy) ensuring the quality of reported identifications and showing overall data consistency. Consequently, our in-house database emerges as a potent tool, providing reliable annotations using only a single-phase lipid extraction method and one analytical platform.

Our research marks a new step in lipidomics, providing a meticulous and comprehensive characterization of the SRM 1950 human plasma reference material. We have achieved an unprecedented balance between coverage of the lipidome and confidence in the annotation, culminating in the generation of a lipid database comprising 592 individual lipid molecular species. The data curation strategy we have applied for the generation of our database has strongly relied on how state-of-the-art knowledge on manual inspection of the data can be applied to drastically reduce the false positive annotation rate while minimizing signal redundancy. It is important to highlight that we have not foregone the key role of software support for annotating and data processing. By integrating all pieces of information, we have provided consistency at three layers —molecular lipid species, lipid subclass, and whole lipidome database—, setting a new standard for accuracy in the annotation and providing a resource to aid in the complexity of untargeted lipidomics. Notably, our study is the first SRM 1950 characterization to incorporate adduct hierarchy for the improvement of data quality.

The use of a one-phase extraction method with direct application and a short LC-MS method —19 min per sample and polarity— allowed high-throughput analysis of the lipidome. By analyzing the profile of healthy individuals, we demonstrated that integrating our database in our lipidomic workflow ultimately constitutes a fast and reliable approach that bypasses the conventional challenges in both data processing and curation. This not only allowed us to provide valuable baseline data for healthy controls, but also facilitates future discovery and clinical lipidomic investigations. In fact, our comprehensive LC-MS lipid database has already been applied in two high-throughput LC-MS-based clinical studies focusing on COVID-19 (22) and Long COVID (52), yielding notable outcomes and illustrating the broad impact and significance of the application of this novel resource in advancing both basic research and clinical applications in lipidomics.

Finally, the LC-QTOF-MS methodology employed in this investigation, in conjunction with our database, facilitates untargeted high-resolution mass spectrometry, enabling the acquisition of MS1 and MS/MS data for unidentified compounds and providing a robust foundation for future expansions in plasma lipidome characterization. The use of underivatized lipid extracts in this platform delivers a comprehensive and reliable overview of the lipidome. However, to address challenges related to the dynamic range and sensitivity inherent in QTOF-MS instrumentation, further refinement may be required. Specifically, compounds present at very low physiological concentrations —such as oxylipins (63, 64)— or with poor fragmentation —such as many bile acids (65)— or lipids with uncommon fatty acyl chains, might benefit from targeted methodologies featuring enhanced sensitivity and specialized sample preparation (62). Additionally, while our current approach offers substantial elucidation of a significant fraction of the plasma lipidome focusing on a widely employed analytical platform and a method runtime suited for high-throughput analysis, further characterization of NIST SRM 1950 could be improved by incorporating ion mobility mass spectrometry (IM-MS) for resolving isomeric overlapping or to overcome the co-elution observed in TG (57).

Ultimately, the high confidence in the annotations present in our newly developed database contributes to facilitate further characterization of SRM 1950, as the comprehensive quantitative distribution and relationships of lipids in this biological matrix remains to be determined by future research studies leading toward the construction of an MS-based lipidomics atlas for the human plasma lipidome (41).

## Data availability

All data described in the article is contained in the article and its Supporting Information except for raw MS and MS/MS data, that have been deposited to the Metabolomics Workbench and are available at NIH Common Fund's National Metabolomics Data Repository (NMDR) Web site, where it has been assigned Study ID ST003514. The data can be accessed directly *via* its Project DOI: 10.21228/M8JK0Q.

## Supplemental data

This article contains supplemental data (66, 67, 68, 69).

## Conflict of Interest

The authors declare that they have no conflicts of interest with the contents of this article.
