# Supplementary material for: Highly reliable LC-MS lipidomics database for efficient human plasma profiling based on NIST SRM 1950
Source: J Lipid Res. 2024 Oct 10;65(11):100671. doi: 10.1016/j.jlr.2024.100671 (PMC11607663; doi:10.1016/j.jlr.2024.100671)
Supplement: Supporting Information [file mmc13.docx]

**Highly reliable LC-MS lipidomics database for efficient human plasma profiling based on NIST SRM 1950**

Sara Martínez^1†^, Miguel Fernández-García^1,2†^, Sara Londoño-Osorio^1^, Coral Barbas^1*^, Ana Gradillas^1^*

^1^Centro de Metabolómica y Bioanálisis (CEMBIO), Facultad de Farmacia, Universidad San Pablo-CEU, CEU Universities, Urbanización Montepríncipe, 28660 Boadilla del Monte, Madrid, Spain.

^2^Departamento de Ciencias Médicas Básicas, Facultad de Medicina, Universidad San Pablo-CEU, CEU Universities, Urbanización Montepríncipe, 28660 Boadilla del Monte, Madrid, Spain.

^†^These authors contributed equally to this work.

*Corresponding author: Coral Barbas: cbarbas@ceu.es

*Corresponding author: Ana Gradillas: [gradini@ceu.es](mailto:gradini@ceu.es)

**Contact information for corresponding author:** Ana Gradillas, Tlf: (+34) 91 3510496, [gradini@ceu.es](mailto:gradini@ceu.es)

Centro de Metabolómica y Bioanálisis (CEMBIO), Facultad de Farmacia, Universidad San Pablo-CEU, CEU Universities, Urbanización Montepríncipe, 28660 Boadilla del Monte, Madrid, Spain.

**Short title:** Increased-Quality Human Plasma LC-MS Lipidomic Database

**Funding sources:** This research was funded by the Ministry of Science and Innovation of Spain (MICINN) and the European Regional Development Fund FEDER, grant number PID2021-122490NB-I00 and La Caixa Foundation, grant number 01-09-2018-01-09-2020.

**Abbreviations:**

AHRL, Adduct Hierarchy Richeness Level; AJS, Agilent Jet Stream; CAR, Carnitine; CCS, Collision Cross Section; CE, Cholesteryl Esther; Cer, Ceramide, FC, Cholesterol; cQualForm, Consensus Qualitative Adduct Formula; cSQuantForm, Consensus Semiquantitative Adduct Formula; DDA, Data-Dependent Acquisition; DG, Diglycerides; DUFA, Di-unsaturated Fatty Acids; ECN, Equivalent Carbon Number; EIC, Extracted Ion Chromatogram; FAC, Fatty Acyl/Alkyl/Alkenyl Chain; GL, Glycerolipid; GP, Glycerophospholipid; HexCer, Hexosyl Ceramide; HRMS, High Resolution Mass Spectrometry; ISF, In-source Fragmentation; ISTD, Internal Standard; KMD, Kendrick Mass Defect; LPC, Lysoglycerophosphocholine, LPC O/P, Alkyl-/Alkenyl- Lysoglycerophosphocholine; LPE, Lysoglycerophosphoethanolamine; LPE O/P, Alkyl-/Alkenyl- Lysoglycerophosphoethanolamine; LPI, Lysoglycerophosphoinositol; LSI, Lipid Standard Initiative; NIST, National Institute of Standards and Technology; NL, Neutral Loss; PC, Glycerophoshphocholine; PC O/P, Alkyl-/Alkenyl- Glycerophosphocholine; PE, Glycerophosphoethanolamine; PE O/P, Alkyl-/Alkenyl- Glycerophosphoethanolamine; PI, Glycerophosphoinositol; QA, Quality Assurance; QC, Quality Control; QM, Quality Management; RM, Reference Material; RP, Reverse Phase; RefLAS, Reference Lipid Annotation Set; RT, Retention Time; SFA, Saturated Fatty Acid; SP, Sphingolipid; SRM, Standard Reference Material, ST, Sterol; TFE, Targeted Feature Extraction; TG, Triglycerides; TUS, Total Useful Signal.

**Table of contents**

| **Additional experimental details** | |
| --- | --- |
| Solvents and reagents. | **S5** |
| Pre-analytics. | **S5** |
| Human plasma reference material information. | **S5** |
| SRM 1950 plasma treatment and lipid extraction. | **S5** |
| Recovery determination. | **S5 – S6** |
| Analytical conditions during RP-UHPLC-ESI-QTOF-MS analysis. | **S7 – S8** |
| Quality Assurance (QA)/Quality Control (QC) analysis. | **S8** |
| Pipeline for the Construction of the In-house Lipid LC-MS Database. | **S8 – S14** |
| Summary pipeline for the Construction of the In-house Lipid LC-MS Database | **S15 – S16** |
| Data pre-processing. | **S17 – S19** |
| **Supporting Tables** | |
| **Table S1.** Database comparison with previous reported results. | **Excel file 1** |
| **Table S2.** In-house SRM 1950 lipid database. | **Excel file 2** |
| **Table S3.** Recovery determination. | **Excel file 3** |
| **Table S4.** Final concentrations of each mixture component of the SPLASH^™^ LIPIDOMIX^®^ C17-sphinganine and palmitic acid d-31 after sample treatment. | **Excel file 4** |
| **Table S5.** Criteria for MS/MS Reference Lipid Annotation Set (RefLAS) lipid species selection. | **Excel file 5** |
| **Table S6.** TFE results in NIST SRM 1950 sample. | **Excel file 6** |
| **Table S7.** Orthogonal properties for quality assessment of the database. | **Excel file 7** |
| **Table S8.** Analysis of TFE results. Correlation values comparing healthy volunteers *vs* NIST SRM 1950 and statistical analysis of average number of carbons and unsaturation degree within lipid species. | **Excel file 8** |
| **Table S9.** Oxylipin standard database used for TFE. | **Excel file 9** |
| **Table S10.** Abundance-corrected average number of carbon atoms from lipid chains within all lipid subclasses in healthy volunteers calculated with TFE results. Average of all individuals and standard deviation. | **Excel file 10** |
| **Table S11.** Abundance-corrected average unsaturation degree from lipid chains of each subclass in all healthy volunteers calculated with TFE results. Average of all individuals and standard deviation. | **Excel file 11** |
| **Table S12.** Qualitative and semiquantitative consensus adduct profile of the RefLAS. | **Excel file 12** |
| **Supporting Figures** | |
| **Figure S1.** Detailed information of the lipid standards used for in-house database annotation validation. | **S20** |
| **Figure S2**. Theoretical *m/z* defect of the experimental adducts found in ESI(+/–). | **S21** |
| **Figure S3**. Data pre-processing approach using a database-based match strategy of the Batch *Targeted Feature Extraction* (TFE) algorithm of MassHunter Profinder (Agilent). | **S22** |
| **Figure S4.** Optimized parameters of the Batch *Targeted Feature Extraction* (TFE) algorithm of Agilent MassHunter Profinder | **S23** |
| **Figure S5.** Input .csv files and adduct selection for each lipid subclass during TFE analysis. | **S24** |
| **Figure S6.** Relative abundance representation of all lipid species –classified per lipid subclass– detected after TFE algorithm. | **S25 – S28** |
| **Figure S7**. Recovery determination by comparing the signal response of the SPLASH LIPIDOMIX spiked into SRM 1950 aliquots, both before and after lipid extraction at three different concentrations –low, medium and high– | **S29** |
| **Figure S8.** Classification of the multitude interferences and redundant information observed during RPLC-ESI-QTOF-MS analysis that can result in false positive annotations. | **S30** |
| **Figure S9.** Example of false-positive annotation given by Lipid Annotator due to the unusual adduct formation ([M+HCOO]^-^) in a PC. | **S31** |
| **Figure S10.** All detected types of isobaric/isomeric overlaps classified according to Lipid Standard Initiative. | **S32** |
| **Figure S11.** Example of in-source fragmentation in CE 18:2 observed during manual inspection. | **S33** |
| **Figure S12**. Isobaric overlap examples observed during manual MS1 inspection: **(A)** Type II isotopic overlap occurs in all double bond series, that is, within a lipid class, the M+2-isotopologue of an unsaturated species overlaps M+0 of species with one double bond less (effect illustrated for sphingomyelins). **(B)** Type II isotopic overlap and various adduct ions isobars within lipid classes (effect illustrated for lysoglycerophospholipids). | **S34** |
| **Figure S13.** Isomeric overlap: selected example observed during MS/MS manual inspection for acyl length chain isomers illustrated for diacylglycerophosphocholines. | **S35** |
| **Figure S14.** Isomeric overlap: selected example observed during MS/MS manual inspection for functional group isomers illustrated for lysoglycerophosphocholines and lysoglycerophosphoethanolamines. | **S36** |
| **Figure S15.** Isomeric overlap: selected example observed during MS/MS manual inspection for sn-position isomers illustrated for lysoglycerophosphocholines. | **S37** |
| **Figure S16.** Selected *sn*-position isomeric overlap observed in diacyl glycerophospholipids containing saturated fatty acids during MS/MS manual inspection. | **S38-39** |
| **Figure S17.** Kendrick mass defect (KMD) vs RT plot showing lipid series in SRM 1950 and mobile phase composition over the chromatographic separation method –mobile phases composition: I = isocratic elution; G = gradient elution– grouping lipid subclasses. | **S40** |
| **Figure S18.** Representation of the correlation between retention time and LogD values predicted by Chemicalize for representative lipids bearing identical C16:0 FAC. Correlations were performed between ranks **(A)** and absolute values **(B).** | **S41** |
| **Figure S19.** RT mapping –among all lipid subclasses detected– for lipid elution order assessment in ESI(+/–) of the sum composition. | **S42 – S44** |
| **Figure S20.** RT shift according to the 95% confidence interval (CI) for each lipid species annotated in NIST SRM 1950 considering the 5 technical replicates of the sample. | **S45** |
| **Figure S21.** Comparison of the lipid database with already reported results. | **S46** |

**Additional Experimental Details**

**Solvents and reagents**

Reverse‐osmosed ultrapure water, used to prepare all the aqueous solutions, was obtained in-house from a Milli‐Qplus185 system (Millipore, Billerica, MA, USA). Methanol (MeOH), acetonitrile (ACN), and isopropanol (IPA), all LC-MS grade, were obtained from Fisher Scientific (Pennsylvania, USA). HPLC grade methyl *tert*-butyl ether (MTBE), ammonium fluoride (NH_4_F) (ACS reagent, ≥ 98%), chloroform (CHCl_3_) was purchased from Sigma‐Aldrich (Steinheim, Germany). Analytical grade ammonia solution (28%, GPR RECTAPUR^®^) and acetic acid glacial (AnalaR^®^ NORMAPUR^®^) were obtained from VWR Chemicals (Pennsylvania, USA).

**Pre-analytics**

SRM 1950 was delivered frozen in 1 mL tubes and stored at –80 ºC until further processing. Prior to lipid extraction, plasma was thawed on ice for 1 h with subsequent vortexing. Plasma was aliquoted in 50 µL aliquots and stored at –80 ºC.

**Human Plasma Sample**

SRM 1950 was used as a sample for the development of the lipid database. The certificate of analysis is available at [www.nist.gov/srm](http://www.nist.gov/srm) and offers a comprehensive description of the material. As a summary, this plasma was derived from a pool of 100 fasting individuals aged 40-50 years, strategically chosen to represent the average demographic composition of the US population in terms of race, sex, and health, excluding extreme health cohorts (1).

**SRM 1950 plasma treatment and lipid extraction**

Three replicates of SRM 1950 were processed through a deproteinization and lipid extraction method using an all-in-one single extraction method based on a solvent mixture composed of MeOH/MTBE/CHCl_3_ (MMC) (2) (4:3:3, v/v/v) to cover the isolation of nonpolar lipids. Lipid extraction protocol involved first, thawing the sample on ice followed by homogenization by vortexing for 2 min. Then, 50 μL of plasma sample was mixed with 800 μL of the solvent mixture containing the ISTD: 2.3 ppm of C17-sphinganine and 4.6 ppm of deuterated (d-31) palmitic acid. In addition, 20 μL of the SPLASH^™^ LIPIDOMIX^®^ were added to the sample and final concentrations of each mixture component are shown in Supplemental Table S4. The resulting mixture was vortexed for 20 min at room temperature followed by sample centrifugation at 16,100 x *g* for 10 min at 15 °C. Finally, 300 μL of the resulting supernatant were transferred to LC Chromacol (Thermo Fisher Scientific, Madrid, Spain) vials with insert and centrifuged at 16,100 x *g* for 5 min at 15 °C prior to the analysis. Blank solutions were prepared containing only H_2_O and the solvent mixture, and the same procedure was followed.

**Recovery determination**

The SPLASH^®^ LIPIDOMIX^®^, used as ISTD for sample analysis, was also employed to determine extraction recovery as a set of exogenous compounds representing different lipid subclasses. Recovery was assessed by comparing the signal response of the SPLASH^®^ LIPIDOMIX^®^ spiked into SRM 1950 aliquots, both before and after lipid extraction at three different concentrations –low, medium, and high– with three sample replicates and three injections per sample. Final concentrations of each deuterium-labeled lipid specie in each case are provided in Supplemental Table S3.

Extraction recovery was calculated following *Eq*(1) and all data is available in Supplemental Table S3.

$$\% Recovery= \frac{average area before lipid extraction}{average area after lipid extraction}\cdot100 Eq(1)$$

**Analytical Conditions during RP-UHPLC-ESI(+/–)-QTOF-MS Analysis.**

SRM 1950 was analyzed using an Agilent 1290 Infinity II UHPLC system coupled to an Agilent 6545 Quadrupole Time-of-Flight (QTOF) Mass Spectrometer (MS) equipped with dual Agilent Jet Stream (AJS) Electrospray ion source (ESI). For the analysis, an optimized method already reported in previous lipidomic studies was used (3, 4).

**Liquid Chromatography**

SRM 1950 replicates were injected in positive and negative ionization modes with volumes of 4 μL and 5 μL respectively using an Agilent 1290 Infinity II Multisampler system kept at 15 °C to maintain compounds stability and prevent lipid precipitation. An Agilent InfinityLab Poroshell 120 EC-C18 (100 mm × 3 mm x 2.7 μm) reversed phase column and guard column (Agilent InfinityLab Poroshell 120 EC-C18, 3.0 × 5 mm, 2.7 μm) were used for lipid separation and temperature was maintained at 50 °C. The mobile phases used for both ionization modes were (A) 10 mM CH_3_COONH_4_ and 0.2 mM NH_4_F in H_2_O/MeOH (9:1, v/v) and (B) 10 mM CH_3_COONH_4_ and 0.2 mM NH_4_F in ACN/MeOH/IPA (2:3:5, v/v/v). The flow rate was set at 0.6 mL/min. The gradient during the chromatographic analysis began with 70% of B at 0 – 1 min, 86% at 3.5 – 10 min, 100% B at 11 – 17.00 min. The initial conditions were restored by min 17.10 followed by a 1.90 min re-equilibration, leading to a total running time of 19 min. The strategy used for multiwash involved a mixture of MeOH/IPA (1:1, v/v) and a wash time of 15 s, followed by a mixture of A/B (30:70, v/v) to assist in reaching the initial conditions.

**Mass Spectrometry**

The Agilent 6545 QTOF-MS equipped with a AJS ESI ion source was set with the following parameters: 175 V fragmentor, 65 V skimmer, 3500 V capillary voltage, 750 V octopole radio frequency voltage, 11 L/min nebulizer gas flow, 290 °C gas temperature, 40 psi nebulizer gas pressure, 11 L/min sheath gas flow, and 370 °C sheath gas temperature.

A solution consisting of two reference mass compounds was infused to the system throughout the whole analysis via an Agilent 1260 Iso Pump at a flow rate of 1 mL/min and with a split ratio of 1:100 for mass correction. The reference masses used were *m/z* 121.0509 (purine detected as [C_5_H_4_N_4_+H]^+^) and *m/z* 922.0098 (HP-0921 detected as [C_18_H_18_O_6_N_3_P_3_F_24_+H]^+^) for ESI(+) and *m/z* 119.0363 (purine detected as [C_5_H_4_N_4_ - H]^-^) and *m/z* 1033.9881 (HP-0921 detected as [C_18_H_18_O_6_N_3_P_3_F_24_+CF_3_COOH-H]^-^) for ESI(–).

Data acquisition was always done - separately for positive and negative ionization modes - using MassHunter Workstation Software LC-MS Data Acquisition v B.09.00 (Agilent Technologies, Waldbronn, Germany).

The MS operated in full scan mode - MS1 level-, was scanned from *m/z* 40 – 1700 at a scan rate of 3 spectra/s.

Then, ten automated iterative-MS/MS were run using data-dependent acquisition (DDA) mode with an automate iterative exclusion (IE) method (5). The acquisition parameters were set following Defossez, E. et al guidelines (6):

1. Scan rate of 3 spectra/s and 3 precursors per cycle.
2. Threshold at 5000 counts and 0.001 % of MS/MS threshold.
3. Mass range scan from *m/z* 40 – 1700.
4. An exclusion list (5) was performed to prevent references masses, noise, and artifacts - non sample related - and detected from blank samples and it was introduced in the method.
5. The collision energy of the first five iterative-MS/MS runs was set at 20 eV, and the remaining five runs were conducted at 40 eV.
6. Isolation window for precursor selection was set as narrow width (∼ 1.3 amu).
7. Isotope exclusion function filter was applied.
8. Data mode in both ionization modes: centroid

**Quality Assurance (QA)/Quality Control (QC) analysis**

Quality management (QM) was consistently maintained throughout the analysis process, encompassing pre-analysis, during analysis, and post-analysis phases. To ensure effective QM, we adhered to the mQACC (Metabolomics Quality Assurance (QA) and Quality Control (QC) Consortium) guidelines (7).

The sample replicates were injected after ten injections of an independent replicate created simultaneously and equal to the sample, to equilibrate the column. Blank samples were prepared containing only the solvent mixture, MeOH/MTBE/CH_3_Cl (4:3:3, v/v/v). Two non-injected blanks consisting of the mobile phases were analyzed prior to the sequence while two solvent blanks were injected at the beginning and end of the sequence to subtract the background signals. Through the analysis of both types of blank samples, it was feasible to pinpoint and isolate any potential sources of contamination. Blank samples followed the same preparation steps as experimental samples.

MassHunter Qualitative Analysis software (B.10.00, Agilent Technologies, Santa Clara, USA) was used to verify system mass accuracy, data quality, and sample reproducibility by the generation of equipment pressure overlays and the evaluation of Extracted Ion Chromatograms (EIC) of reference masses and ISTD.

**Pipeline for the Construction of an In-house Lipid LC-MS Database. Six main Steps for Manual Annotation Quality Assessment**

MassHunter Qualitative Analysis B.10.00 software by Agilent technologies, was used for all the manual curation processes carried out on all HRMS.raw data during the structural lipid characterization.

An in-house Lipid LC-MS Database, based on SRM 1950, was built with high confident lipid annotation by following the steps detailed below:

***Step 1. Generation of a Reference Lipid Annotation Set (RefLAS).***

***Software-assisted lipid annotation***

Iterative DDA MS/MS datasets obtained from LC-MS analysis were underwent screening, to identify spectra compatible with specific lipid molecular species, using three independent software tools to identify lipid molecular species based on similar previous strategies (8). The software used were the vendor software Agilent MassHunter Lipid Annotator (9) (Agilent Technologies, Inc.), and two open-source software, MS-DIAL 4 (10) (Riken Center for Integrative Medical Sciences) and Lipid Hunter 2 (11) (University of Leipzig).

1. All iterative MS/MS (.d) raw files were converted to open-source (.abf) and (.mzML) formats using ABF (Analysis Base File) Converter and ProteoWizard’s MSConvert respectively for their posterior use in the open-source software MS-DIAL and LipidHunter.
2. Raw iterative MS/MS dataset obtained were analyzed using the three annotation software tools. The parameters for each software were set as follows:
   1. **Lipid Annotator vendor software (.d file):** The unique filter used was Q-Score which was set at greater than 30. For annotation parameters ESI(+) adducts were selected as [M+H]^+^, [M+Na]^+^ and [M+NH_4_]^+^, while ESI(–) adducts included [M-H]^-^, [M+CH_3_COO]^-^. *m/z* tolerance was limited to 20 ppm and both fragment score and total score were required to exceed 30 and 60, respectively.
   2. **MS-DIAL 4 open-source software (.abf file):** Parameters included MS1 and MS/MS tolerance of 0.01 Da and 0.025 Da respectively. The mass-to-charge (*m/z)* range for both MS1 and MS/MS was set as 40 – 1700 with a retention time (RT) range from 0 to 20 min. Minimum peak height was defined as 1,000; with a mass slice width of 0.1 Da. Additionally, smoothing was applied at level 1 scans and the minimum peak width was set to 5 scans. An identification score cut off was established at 70% and adducts ion settings were selected based on Fig. 4.
   3. **Lipid Hunter 2 open-source software (.mzML file):** Parameters were configured with a scan time range from 0 to 20 min and a *m/z* range of 40 – 1700. Mass tolerance was set at 20 ppm for both MS and MS/MS level. A MS intensity threshold of 1,000 counts and a MS/MS intensity threshold of 10 counts were applied. Identifications were filtered based on isotopic score (R > 65) and rank score (R > 60). The FA whitelist used was the default configuration file ‘‘FA Whitelist.xlsx’’.

***Merge of software annotation results:***

Subsequently, the annotation tables outputted from these software were merged, and only the lipids fulfilling the reported inclusion criteria (Supplemental Table S5) were retained as potentially true positive annotations requiring further validation described below and following a similar strategy to the already previously reported (8, 12):

1. Redundant annotations, for example different lipid ion adducts or clusters of the same molecular species were removed.
2. Results obtained from the three software were cross-matched and filtered separately for positive and negative analysis, in an Excel file. Briefly, candidate matching at least in two of the three annotation software was considered as correctly annotated; if only a lipid specie was found by one software and the other two do not yield any compound for that molecular feature, total score will be needed. If total score > 80%, the lipid specie will be considered annotated, if < 80% manual inspection using Agilent MassHunter Qualitative analysis was done considering mass accuracy (maximum mass tolerance ±10 ppm), RT accuracy (maximum RT tolerance ±0.3 min), specific fragment ions, adduct formation pattern and isotopic pattern distribution, leading to the correct annotation of individual lipid species. When only sum composition was given by the software, additional manual MS/MS inspection was performed to assess the structural composition.

***Assessment of the software-assisted lipid annotation: Manual MS/MS spectra inspection***

Then, a manual inspection of every fragmentation pattern was carried out for lipid assignment validation, based on two main inputs: the precursor ion mass and the fragmentation pattern in order to:

1. Ensure that software-assisted lipid annotation was based on high-quality MS/MS spectra, taking into account that automated DDA-MS/MS spectra often produce highly convoluted spectra –“chimeric” MS/MS spectra that contain fragments from multiple co-eluted precursor ions (13)–, all those contaminated spectra (especially for low abundant lipids and/or highly co-eluted lipids such us TG) were rejected for accurate annotation, and only those with high-quality are trusted (13).
2. Validate annotation accuracy with fragments ions corresponding to specific lipid subclass or molecular species fragments that were assessed in ESI(+/–)-MS/MS following:

- guidelines provided by the Lipidomics Standard Initiative resources (14)

<https://lipidomicstandards.org>

- and detailed description given by Lange M et al.(8) as supplemental information for manual lipid annotation described at <https://github.com/SysMedOs/AdipoAtlasScripts/blob/main/LipidIdentification/AdipoAtlas_Lipid_Identification_Manual.pdf>:

1. Solve **functional isomeric interferences** (e.g. PC *vs* PE lipid subclasses).
   1. by checking specific fragments ions related to the head group or specific neutral losses (8)
2. Solve **chain isomeric overlaps**:
   1. by checking in ESI(–)-MS/MS the specific fragment ions for assignment of the fatty acyl chains (FAC), (e.g. PC 36:2: which can lead to several chain isomers such as PC 18:1_18:1 or PC 16:0_20:2 among others depending on FAC composition).
3. Solve ***sn*-positional isomeric overlaps**:
   1. by checking in ESI(+)-MS/MS the intensity ratio of characteristic fragment ions such as LPC *sn-*1 and *sn-*2 position (e.g., LPC 18:0/0:0 and LPC 0:0/18:0); and by checking in ESI(–)-MS/MS the intensity ratio of fragments ions related to the positions of FAC such as PE/PC/PI (e.g., FAC with a higher intensity rate corresponds to *sn*2 position being PX *sn-*1/*sn-*2). Detailed description given by Lange M et al. as supplemental information for manual lipid annotation described at (8) and by Alcoriza-Balaguer M.S. et al as supplemental information at (15)
   2. Distinction among *sn*-1, *sn*-2 and *sn*-3, positions in TG species couldn’t be identified due to highly convoluted MS/MS spectra.

***Assessment of isobaric and isomeric overlaps***

Across all steps one important aspect to achieve correct identification is the consideration of isobaric and isomeric overlaps inherent in lipids, Supplemental Fig. S10.

On the one hand, the so-called type II isobaric overlap occurring in double bond series (16, 17) within the lipid class was always evaluated when the M + 2-isotopologue, [M+^13^C_2_+H]^+^, of an unsaturated species overlaps M + 0 of species with one double bond less.

In parallel, the presence of isobaric overlap resulting from various adduct ions –within and between lipid classes– was taken into account (see <https://lipidomicstandards.org/isobaric-overlap/>) (see examples in Supplemental Fig. S12). On the other hand, the addition of unresolved isomeric interferences reduced positive and negative lipid identities (18) (Supplemental Fig. S13-S16).

***Assessment of RP-LC elution profile (RT mapping)***

The first sub-set of lipids, obtained from the above software-assisted MS/MS annotations, was further validated by performing comprehensive evaluation of orthogonal data such as the RT behavior across the 20 min of chromatographic run. In addition, multiple related ion-species generated for the same lipid were inspected –adducts, clusters of non-volatile salts, and in-source fragments grouped based on RT–.

Thus, MS1 data were underwent screening to gain additional reliability and consistency in the annotations by performing the following:

1. Assessment of each lipid elution order in ESI(+/–) by plotting the RT of a given lipid specie against its sum composition carbon number within each unsaturation serie (RT mapping), using R (v. 4.3.0) and the package *ggplot2*. Process where we also evaluated the consistency with ISTD from the same lipid subclass (Supplemental Fig. S19) (16). The use of RT mapping plot allows visual inspection of elution order of homologous series of the same lipid class depending on relative carbon number and/or relative unsaturation number (16).

Annotations not fulfilling points **i-vi** were rejected as false positives. At the end of this curation process a **Reference Lipid Annotation Set (RefLAS)** of 289 lipids was generated. To benchmark the RefLAS data, exogenous ISTD was used for reliable lipid annotations comparison on orthogonal information within the same lipid subclass (Supplemental Table S7).

***Validation of the annotations against lipid standards***

Some annotations were validated against **31 lipid standards** from 10 lipid subclasses between both ionization modes (when available for the lipid subclass). Thus, validation was performed by spiking SRM 1950. Using MS1 scanning mode and matching exact mass and RT all the initial annotations were confirmed.

***Step 2. Evaluation of the qualitative and semiquantitative adduct and cluster profile and in-source fragmentation during electrospray ionization (ESI).***

Having defined our panel of 289 lipids belonging to **RefLAS**, Supplemental Table S7, the presence/absence of each evaluated adduct and cluster was characterized as well as determined their semiquantitative order of appearance in ESI(+/–) (e.g., [M+H]^+^ > [M+Na]^+^ > [M+K]^+^) by performing the following:

1. Manual extraction and evaluation of the presence/absence and semiquantitative order of apparition of theoretical *m/z* adducts and clusters of non-volatile salts following generation rules from our method previously described (19). Manual extraction and evaluation of the presence/absence and semiquantitative order of apparition of theoretical *m/z* adducts and clusters of non-volatile salts following generation rules from our method previously described (19).

Theoretical *m/z* adduct calculation was performed following Supplemental Fig. S2. The following adduct ion *m/z* were extracted for each of these annotations:

**ESI(+):** [M+H]^+^, [M+Na]^+^, [M+K]^+^, [M+C_2_H_7_N_2_]^+a^, [M+NH_4_]^+^

**ESI(–):** [M-H]^-^, [M-H+(CH_3_COONa)]^-^, [M+CH_3_COO]^-^, [M+CH_3_COO+(CH_3_COONa)]^-^, [M+CH_3_COO+(CH_3_COONa)_2_]^-^, [M+CH_3_COO+(CH_3_COONa)_3_]^-^, [M+HCOO]^-b^, [M+HCOO+(CH_3_COONa)]^-^, [M+Cl]^-^

^a^ACN from mobile phases can yield acetamidinium cation in ESI(+) producing the unexpected [M+C_2_H_7_N_2_]^+^ adduct (19).

^b^MeOH from mobile phases can be oxidized to formic acid during ESI(–) producing unexpected [M+HCOO]^-^ adducts even when formic acid is not used in the mobile phases, which can lead to false-positive identification. ESI source can be considered as an electrochemical cell and the occurrence of certain unpredictable electrochemical redox reactions may result in several unexpected ions that lead to misannotations (i.e. [PC 36:4+HCOO]^-^ = [PC 35:4+CH_3_COO]^-^ (Supplemental Fig. S9)).

Moreover, acetate salts of LC-MS grade can be contaminated with trace amounts of formate,

1. Assessment of ISF (see <https://lipidomicstandards.org/in-source-fragmentation/>) informative of the lipid subclass to both assist in the annotation quality assessment and to address the impact of unintended in-source fragments on lipid profile characterization (20–22).
2. Subsequently, manual inspection of the height at the apex of the peak of each lipid for the predominant adduct in each lipid subclass was performed. For this purpose, individual extracted ion chromatograms (EICs, ±20 ppm *m/z* extraction width) were extracted by inputting the *m/z* values corresponding to adduct ions determined as order 1 in the adduct hierarchy of their subclass, Fig. 4. For lipid subclasses where more than one adduct ion was scored as order 1, both adduct ions were extracted and peak intensities were summed. Within this process, mass accuracy and RT values were additionally manually corrected.

Both consensus qualitative and consensus semiquantitative adducts and cluster formulas (cQualForm and cSQuantForm respectively) were generated, as well as the adduct hierarchy richness level (AHRL) values obtained from each cSQuantForm in each RefLAS annotation (Supplemental Table S2 and Table S8).

***Step 3. Assessment of Consistency of Non-Reflas Annotations Obtained from Annotation Software with RefLAS.***

1. Then, annotations not accomplishing RefLAS criteria but detected by automated software were iteratively subjected to quality in the annotation assessment of manual MS/MS spectra, and quality assessment of annotation information described in above steps: RT mapping, adduct qualitative information and hierarchy, and presence of ISF. Annotations not accomplishing MS/MS quality criteria, RT mapping or cQualForm consistency with the RefLAS were removed as false positives. Retained annotations were subjected to step (**ix**).

***Step 4.* *MS1-Based Extension of the Lipid Database: Completion of Homologous Series for each Lipid Class.***

A MS1-based database extension of the lipid database was performed by the manual characterization of lipid molecules classified by the homologous lipid series of the same subclass, evaluating consistency in all the pieces of information described in steps 1 and 2.

Annotations not complying consistency with the RefLAS and annotations validated in step (**x**) in terms of mass measurement accuracy (±20 ppm *m/z* extraction width), adduct qualitative formula, and RT –within the expected window based on RT mapping– were excluded as false positives. Validated annotations were also subjected to step (**ix**).

This was performed sequentially, evaluating different types of candidate annotations:

1. Lipids described in the literature or predicted from biological knowledge (23–26). We extracted traces from predicted adducts.
2. Lipids corresponding to gaps in the chemical space defined by our RT mapping plots. We performed a manual gap-filling inspection of RT versus carbon number of the sum composition plots, where individual adduct ion traces of predicted lipid species given the chemical space contour defined by the lipid species present in the plot were extracted.

***Step5. Structural Characterization of the*** ***Fatty Acyl Chains Composition***

Having annotated the lipid subclasses, we further proceeded into more structurally resolves species detailing the fatty acyl chains (FAC) reported by RefLAS and non-RefLAS annotations having associated MS/MS spectra by already reported criteria (8, 27) and manual inspection of “chimeric” MS/MS spectra (13).

For mono- and diacyl/alkyl/alkenyl-*sn*-glycerol series, FAC elucidation was performed by direct assignment from the sum composition when MS/MS data supporting acyl chain annotation was available. Further assessment of FAC annotation was addressed by evaluating in lipids bearing two FAC residues, the effect of carbon number and degree of unsaturation in each FAC (28).

Thanks to the use of intensity ratios between characteristic fragments, elucidation of the *sn*-positions was possible. Attention must be paid to lysogylcerophospolipids, such LPC and LPE, and to glycerolipids such as DG, where interconversion of *sn*-positional isomers occurs during sample handling and preparation. Thus, acyl migration between *sn*-1 and *sn*-2 positions, during analysis, always exists and makes annotations for these lipids more complicated (18).

Having characterized the profile of FAC by MS/MS whenever possible, we ensured consistency in the RT dimension for species with identical sum composition belonging to the lipid subclass for one-FAC containing lipids, diacylGP, and SL. Monoacyl/alkyl/alkenylGP were curated according to that the sn-2 lyso form is less retained than the sn-1 counterpart. DiacylGP were curated using the behavior observed in PC (assuming equal behavior irrespective of the polar head) for compounds having (i) equal FAC unsaturation number but different FAC carbon number, and (ii) different FAC carbon number for equal FAC unsaturation number. SL were curated evaluating effects (i) and (ii) using SM as reference.

***Step 6. Validation by overall evaluation of the lipid database consistency in pieces of information***

After having expanded our lipid library, we iteratively performed a last validation of the data contained in our database in pieces of information which should comply consistency:

1. RT dimension between lipid series, as lipids with distinct functional groups have a well characterized behavior already described for our method which we compared against the predicted logD (Chemicalize) of selected examples bearing 16:0 FA chains.
2. RT mapping compliance within each lipid unsaturation series of each lipid subclass, as performed in step (**vi**)
3. Full compliance of all annotations present in the database in cQualForm.
4. *m/z* consistency evaluation of the predominant adduct ion within each lipid subclass, where we calculated average, standard deviation, and Z-score values for the mass accuracy values. We considered outliers in mass accuracy these compounds which showed a Z-score > 2.
5. Consistency evaluation of all annotations in terms of cSQuantForm and AHRL for low-abundant compounds.

**SUMMARY _ PIPELINE FOR THE CONSTRUCTION OF AN IN-HOUSE LIPID LC-MS DATABASE**

**Step 1.** **GENERATION OF A REFERENCE LIPID ANNOTATION SET (RefLAS)**

***Software-Assisted Lipid Annotation***

**INPUT:** Iterative exclusion DDA-MS/MS datasets = *.d or *.mzmL raw data files

1a. Ion precursor *m/z* tolerance ±10ppm

**OUTPUT**: - Merged table of non-curated annotations

***Evaluation of the software-assisted lipid annotation: Manual MS/MS spectra inspection***

**INPUT:**

2a. Manual MS/MS spectra quality evaluation: assessment of convoluted spectra. Evaluation of isobaric and isomeric overlaps. Determination of the presence of diagnostic fragments >> Defined criteria for subclass and acyl chain composition. >> false positive removal

2c. Qualitative adduct profile assessment >> false positive removal

***RT mapping***

2d. Lipid sum composition assessment - RT elution order compliance within each unsaturation series and with ISTD >> false positive removal

**OUTPUT:** - Compliance level according to each of the pieces of information evaluated

- RefLAS criteria definition for each lipid subclass evaluated

- RefLAS and non-RefLAS assignment for each annotation

***Validation of the annotations against lipid standards***

- Annotation validation by spiking SRM 1950. Using MS1 scanning mode and matching exact mass and RT.

**Step 2. EVALUATION OF THE QUALITATIVE AND SEMIQUANTITATIVE ADDUCT AND CLUSTER PROFILE AND IN-SOURCE FRAGMENTATION DURING ELECTROSPRAY IONIZATION (ESI).**

***Assignment of within-method cQualForm, cSQuantForm and AHRL values, and ISF***

- Manual extraction of RefLAS adduct profile and intensity of the predominant adduct

- Generation of cQualForm based on RefLAS - Generation of cSQuantForm based on RefLAS

- Definition of AHRL levels based on RefLAS

- Determination of literature described ISF in RefLAS annotations

**Step 3. ASSESSMENT OF CONSISTENCY OF NON-REFLAS ANNOTATIONS OBTAINED FROM ANNOTATION SOFTWARE WITH REFLAS**

4a. MS/MS (absence of diagnostic fragments only justified if low abundant) >> false positive removal unless 4b and 4c are accomplished

4b. RT mapping - compliance with RefLAS and ISTD >> false positive removal

4c. Adduct cQualForm - compliance with RefLAS and ISTD >> false positive removal

**OUTPUT:** - dataset of software assisted annotations with false positive removal and consistency evaluation between RefLAS and non RefLAS annotations

**Step 4. MS1-BASED EXTENSION OF THE LIPID DATABASE: COMPLETION OF HOMOLOGOUS SERIES FOR EACH LIPID CLASS**

**Database expansion through raw data search**

5a. Literature-based expansion

5b. Assessment of literature obtained annotations in consistency in pieces of information with

**OUTPUT**: From step 4, according to 4a to 4c evaluation steps

- Gap-filling based expansion of data using RT mapping

- Assesment of gap-filling obtained annotations according to 4a to 4c

**Step 5. STRUCTURAL CHARACTERIZATION OF THE FATTY ACYL CHAINS COMPOSITION**

- Determination of annotation level according to MS/MS

- Evaluation of acyl chain effect on RT for each defined sum composition using PC SM and Cer as reference

**Step 6. VALIDATION BY OVERALL EVALUATION OF THE LIPID DATABASE CONSISTENCY IN PIECES OF INFORMATION**

6a. Required correlation of RT behavior according to the lipid subclass and predicted polarity values**(chol)

6c. Required compliance of RT mapping according to the sum composition for all lipid subclasses

6d. Required compliance of cQualForm for all lipids present in the database

6b. Consistency evaluation within lipid subclass of MS1 *m/z* tolerance of the predominant adduct

6e. Consistency evaluation of all lipid entries in cSQuantForm, and AHRL-intensity

**Data Pre-Processing**

For data pre-processing, the in-house lipid LC-MS^n^ database was used as a template to perform targeted peak extraction based on a molecular formula, mass accuracy and RT-match approach. Thus, MS1 raw data (.d) of the five SRM 1950 replicates obtained through ESI(+/–) analysis were first assessed using MassHunter Qualitative Analysis to ensure MS data quality, (Fig. 1C). Then, peak detection was performed using the Batch Targeted Feature Extraction (TFE) algorithm of MassHunter Profinder software (B. 10.0.2, Agilent Technologies, Santa Clara, USA) that uses an input molecular formula source, a (.csv) containing lipid ID, molecular formula, *m/z*, and RT information to extract lipids from the sample data files using a process referred to as Find Compounds by Formula.

A description of all optimized parameters used in the TFE algorithm, and a schematic illustration of the pipeline is shown in Supplemental Fig. S3 and Fig. S4 and is described in detail below:

**Pre-processing:**

To increase the performance of the database and avoid the software to force adduct detection, the database was subdivided into several input .csv files based on the adduct profile formula of each lipid subclass of the RefLAS.

The main ionization mode was selected for each lipid subclass based on adduct ion intensity. In accordance with previous studies that have evaluated the effect of adduct formation variability on the accurate quantification of compounds (29) we selected in the TFE algorithm the adducts that composed at least the 95% of the total abundance within each lipid subclass. This was performed by manual examination of the three more abundant lipid species of each of the subclasses of the RS using Agilent MassHunter Qualitative B 10.00. Input .csv files and adduct selection for each lipid subclass are shown are shown in Supplemental Fig. S5.

**Profinder Batch Targeted Feature Extraction Algorithm (TFE) Parameters**

The Batch *Targeted Feature Extraction* (TFE) algorithm in MassHunter Profinder was used to extract the features from all files in the LC-MS dataset. The parameter settings optimized and used during data preprocessing are detailed in the following steps and shown in Supplemental Fig. S4:

1. **TFE – Molecular Formula Target:**
   1. Formula source: The specific (.csv) based on the database containing the ID lipid species, molecular formula, RT and monoisotopic mass was uploaded and the values to match were set as mass and RT (RT required), allowing a maximum of one number of matches.
   2. Ion species: For each lipid subclass csv input file, the ionization mode and adducts were selected as shown in Supplemental Fig. S5**.**
   3. Charge state: For ESI(+) the *Common organic (no halogens)* model was selected. For

ESI(–) the model *Common organic molecules* and 1 charge state was selected.

1. **TFE – Matching Tolerances and Scoring**
   1. Formula marching: mass tolerance was set as ±20 ppm and RT tolerance as ±0.5 min. The expected RT was set symmetrically within ±2.50 min and Extracted Ion Chromatogram (EIC) was limited to the extraction range. The possible *m/z* was set as symmetric (ppm) ±35.
   2. Scoring: The isotope abundance and RT score were set as 80 and 99 respectively and other parameters were set as default.
   3. Result filters: Set to warn if score < 60.
2. **EIC Peak Integration and Filtering**
   1. Integration: Integration was set as ‘Agile 2’
   2. Smoothing: Smoothing EIC before integration was selected and the Quadratic/Cubic Savitzky-Golay smoothing function with a function width of 9 points was chosen. Other parameters were set as default.
   3. Peak filters: Peak selection was based only on peak height and no other filters were selected.
   4. Chromatogram format: The chromatogram data format chosen was centroid.
3. **Spectrum Extraction and Centroiding**
   1. Peak spectrum: All parameters were maintained as default.
   2. Centroiding: Peak location filters were established with a maximum spike width of 2 and a required valley of 0.70.
   3. Spectrum format: Spectrum format was configured as centroid.
4. **Post-Processing Filters**
   1. The score (Tgt) was set as ≥ 50 and the minimum filter matches was set to 1 file in at least one sample group.

**Evaluation of the TFE matching accuracy:**

1. After running the method for TFE algorithm, a table with all lipid details (ID, molecular formula, monoisotopic mass, RT (min), corresponding peak area and match score among others) were obtained, as well as the extracted ion chromatogram (EIC) and the MS1 spectra which provided information about the adduct profile and isotopic pattern of all matched lipid species.
2. The initial chromatographic results were manually inspected to ensure that lipids were matched based on high-quality peaks. Thus, we rejected those with poor quality or from background noise level. The evaluation of peak integration was also carried out and refined where appropriate.
3. For annotations with score < 60 the MS spectrum results were manually evaluated to remove those peaks without the reasonable isotopic pattern and improbable adduct distribution –e.g., PC where [PC + Na]^+^ was the detected as the main adduct if the corresponding [PC + H]^+^ ion was not observed, or those TG detected as [TG + Na]^+^, if the corresponding [TG + NH_4_]^+^ ion was not observed–.
4. Manual curation using MassHunter Qualitative Analysis was performed to assess and discard false-positive annotations when isomeric interferences were detected.
5. Mismatching RT and *m/z* tolerance

**Data export**

1. The curated results were exported from Profinder software to a (.csv) and saved as a (.xlsx).
2. ESI(+) and ESI(–) output results were combined, and duplicates were removed based on the abundance and ionization mode in which better ionizes each lipid subclass.
3. Finally, a high-quality identification lipid table comprising all matched lipid was generated. Then, the average of the relative abundance replicates was calculated for further determinations.

After data export, a second TFE analysis was performed using the same optimized parameters and posterior curation criteria in order to reduce the number of false negatives not detected by Profinder software. Specific Agilent (.csv) files were created in ESI(+/–) with the lipid species present in the lipid database not detected in the previous Profinder run and procedure was followed as already described.

**Figure S1.** Detailed information of the lipid standards used for in-house database annotation validation.

**
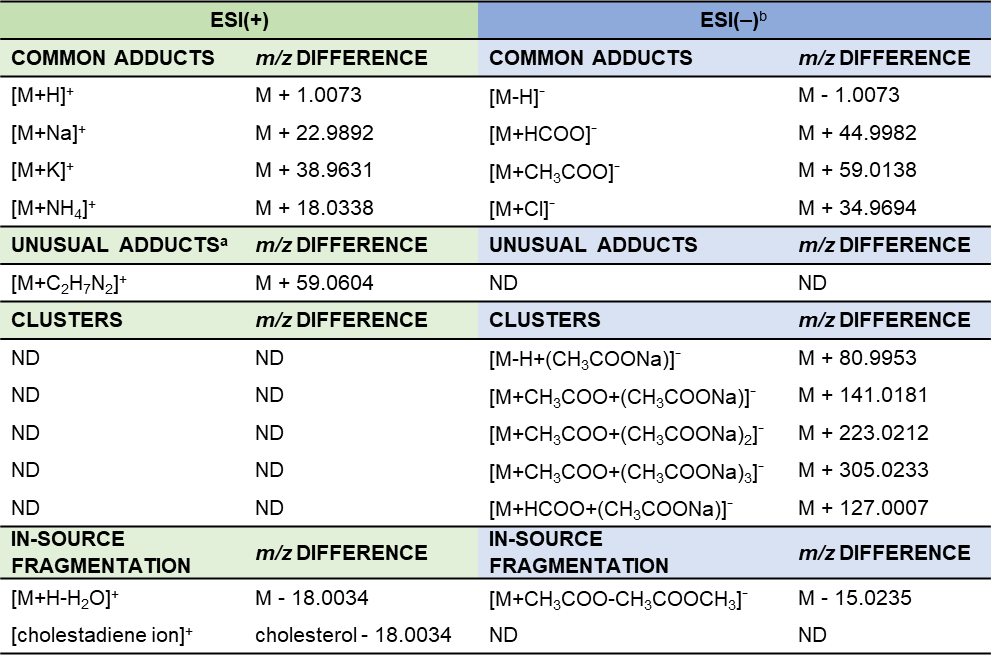
**

**Figure S2.** Theoretical *m/z* difference of the experimental molecular ion adducts, clusters and neutral losses observed in ESI(+/–) mass spectra.

ND = non detected

^a^[M+C_2_H_7_N_2_]^+^ = acetamidinium adduct (19)

^b^Acetate salts of LC-MS grade can be contaminated with trace amounts of formate, which can lead to false-positive identification. Moreover, Methanol from mobile phases can be oxidized to formic acid during electrospray ionization.

^c^Sodium acetate (+ 82.0031 Da) appear as repeating units (n = 1-6) in ESI(–) in LC-MS background.

**Figure S3.** Data pre-processing approach using a database-based match strategy of the Batch *Targeted Feature Extraction* (TFE) algorithm of MassHunter Profinder (Agilent).

**Figure S4.** Optimized parameters of the Batch *Targeted Feature Extraction* (TFE) algorithm of Agilent MassHunter Profinder.


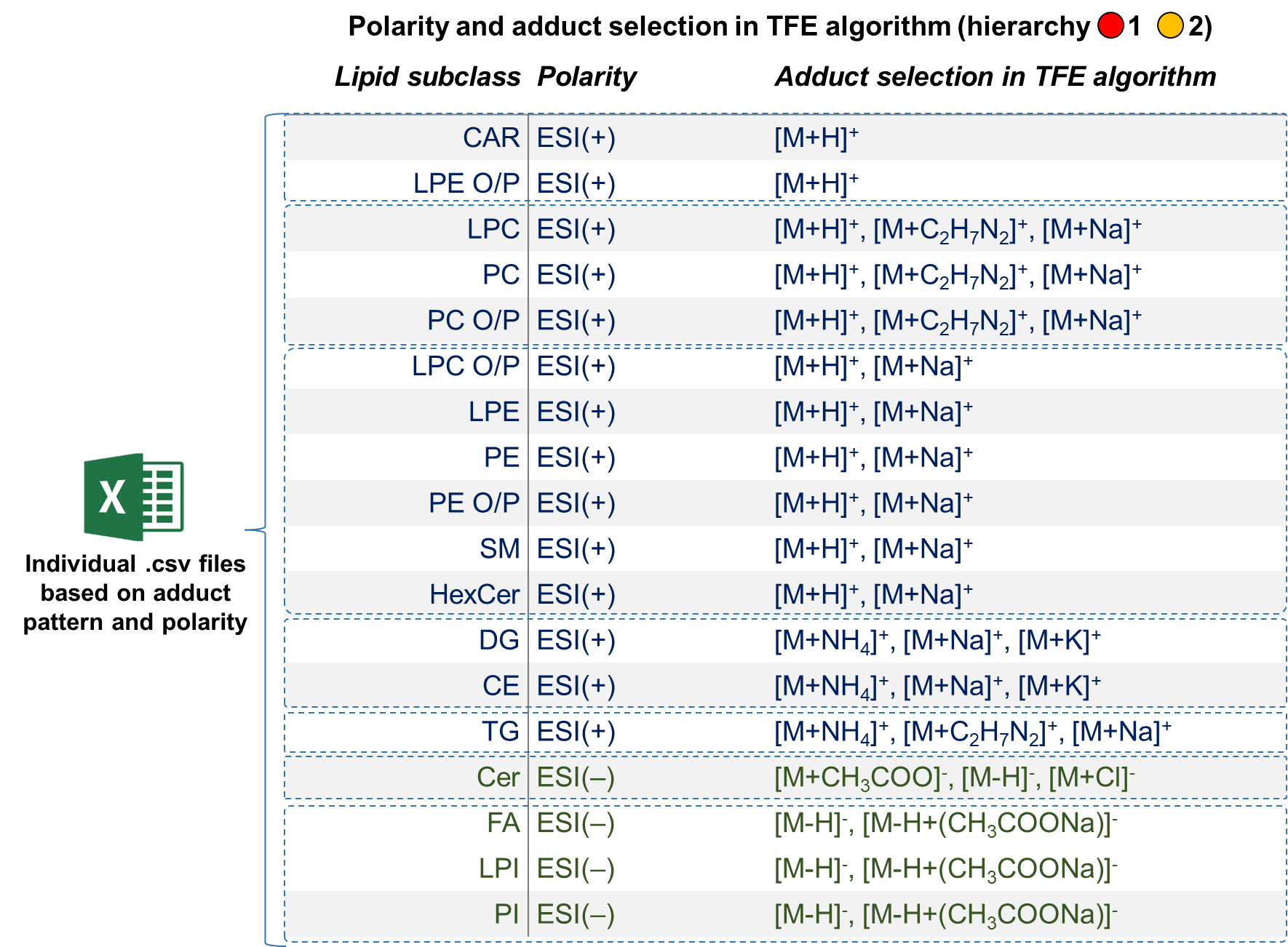


**Figure S5.** Input .csv files and adduct selection for each lipid subclass during TFE analysis.


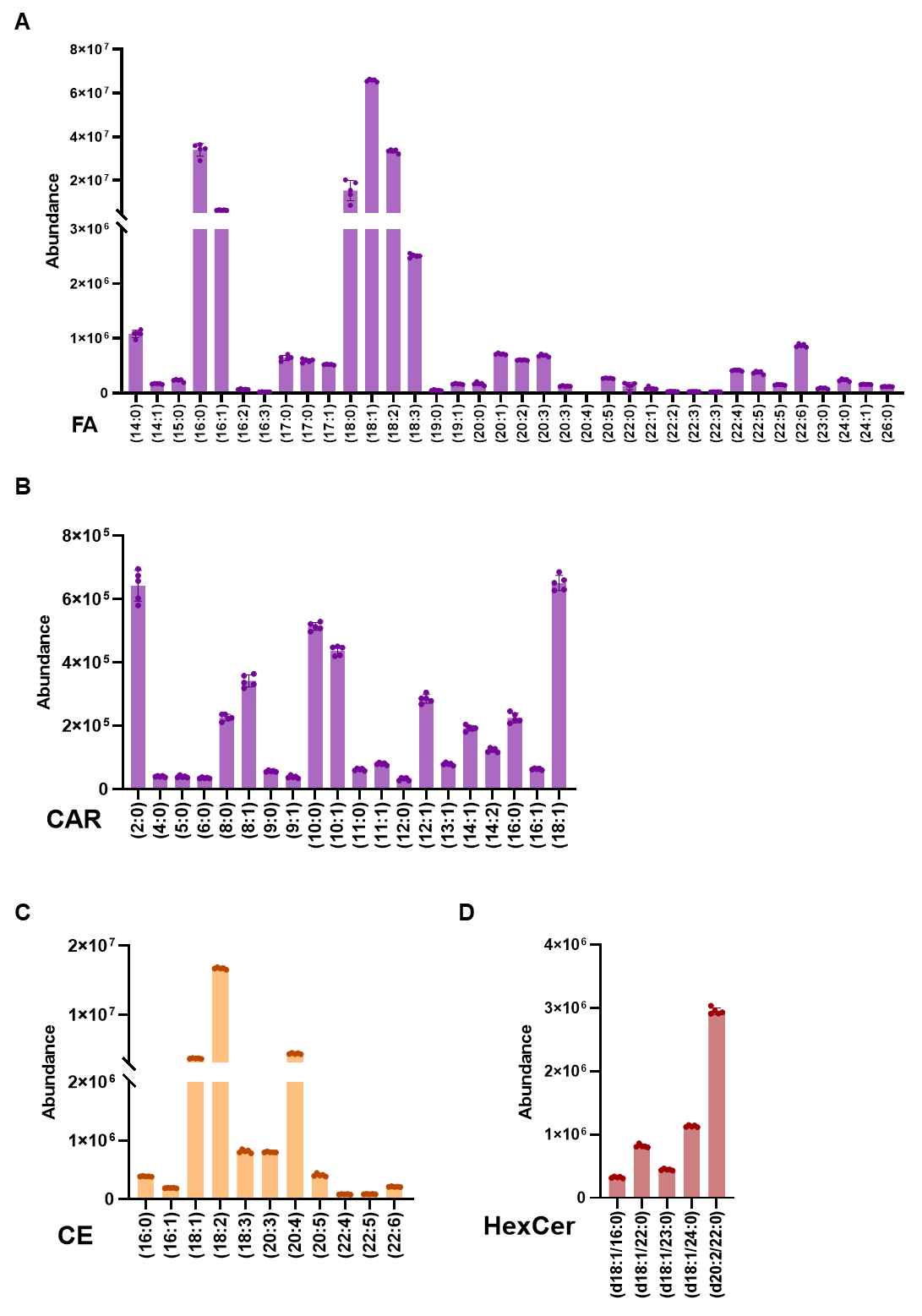


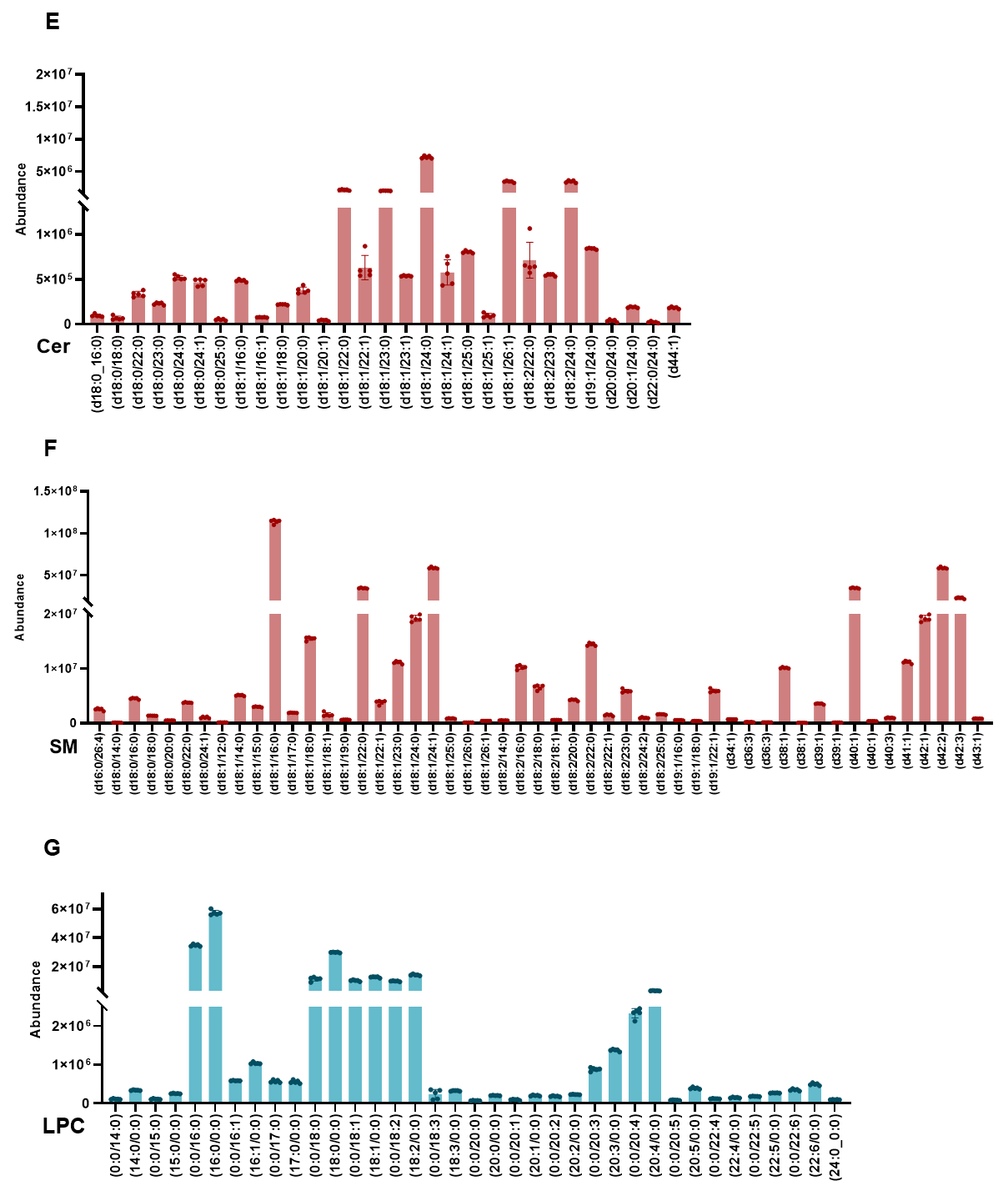


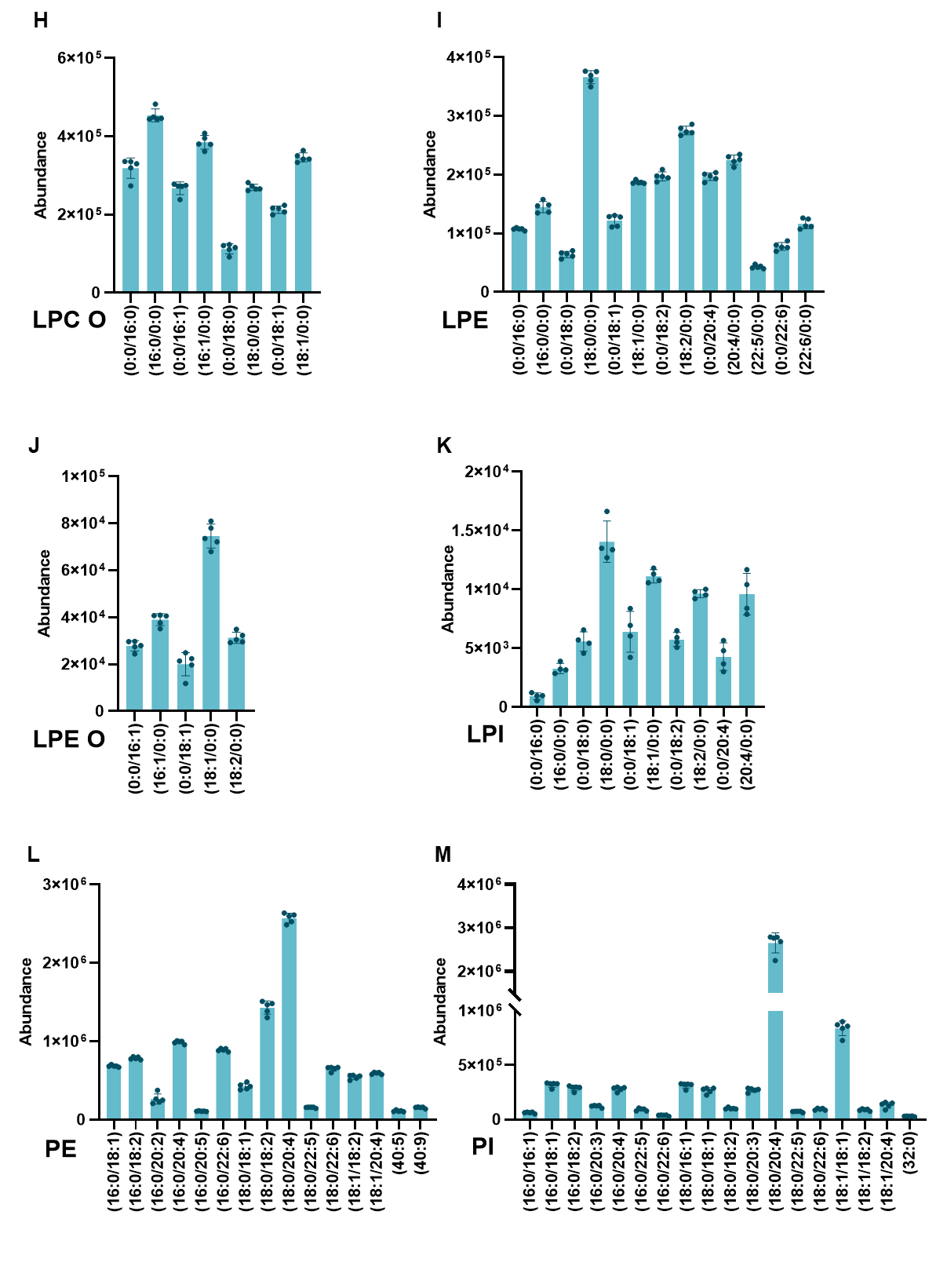


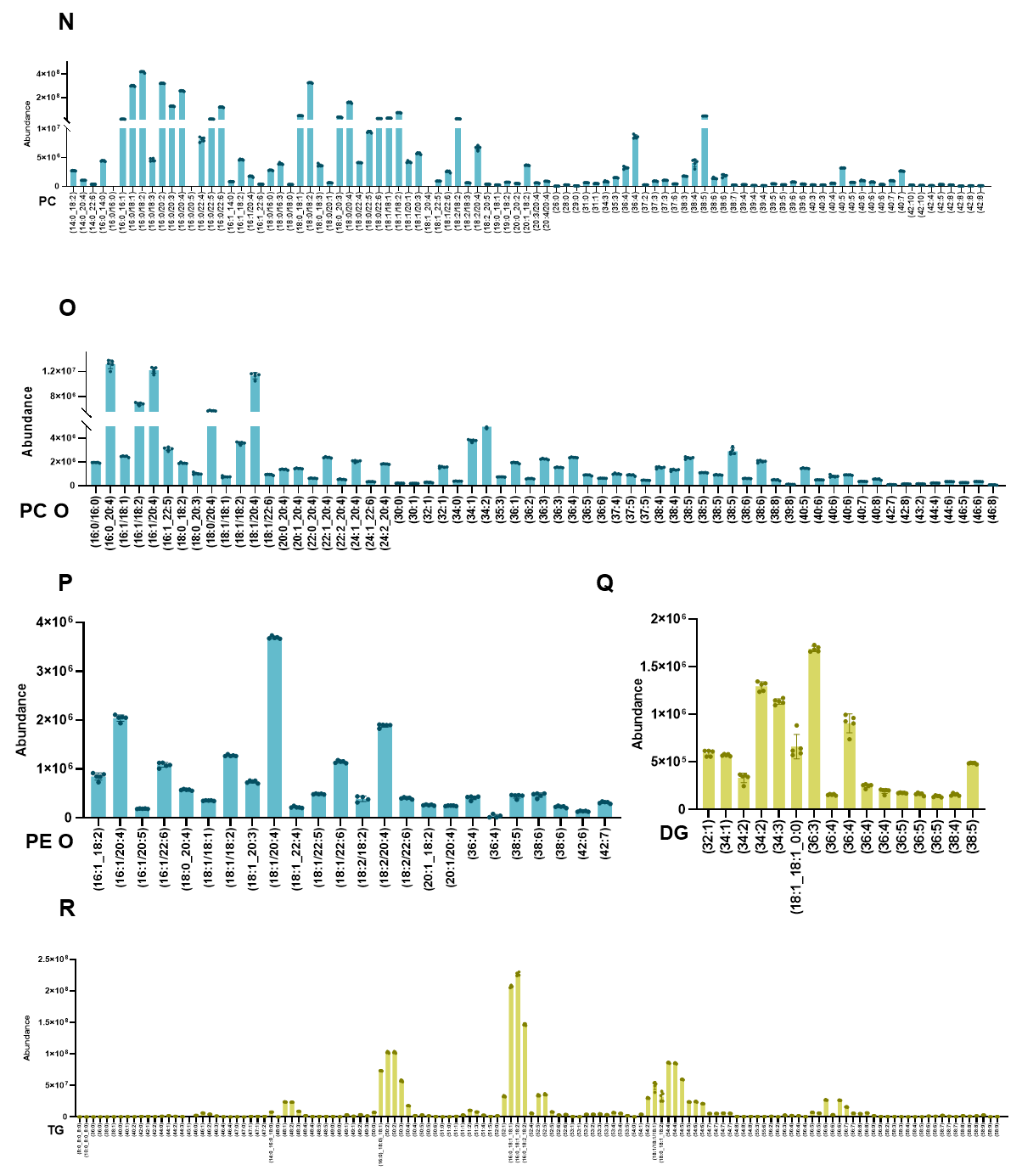


**Figure S6.** Relative abundance representation of all molecular lipid species –classified per lipid subclass– detected after TFE algorithm in SRM 1950 replicates.

*For lipid subclasses LPC O/P, LPE O/P, PC O/P and PE O/P, only the alkyl form (O-) was written as a label.


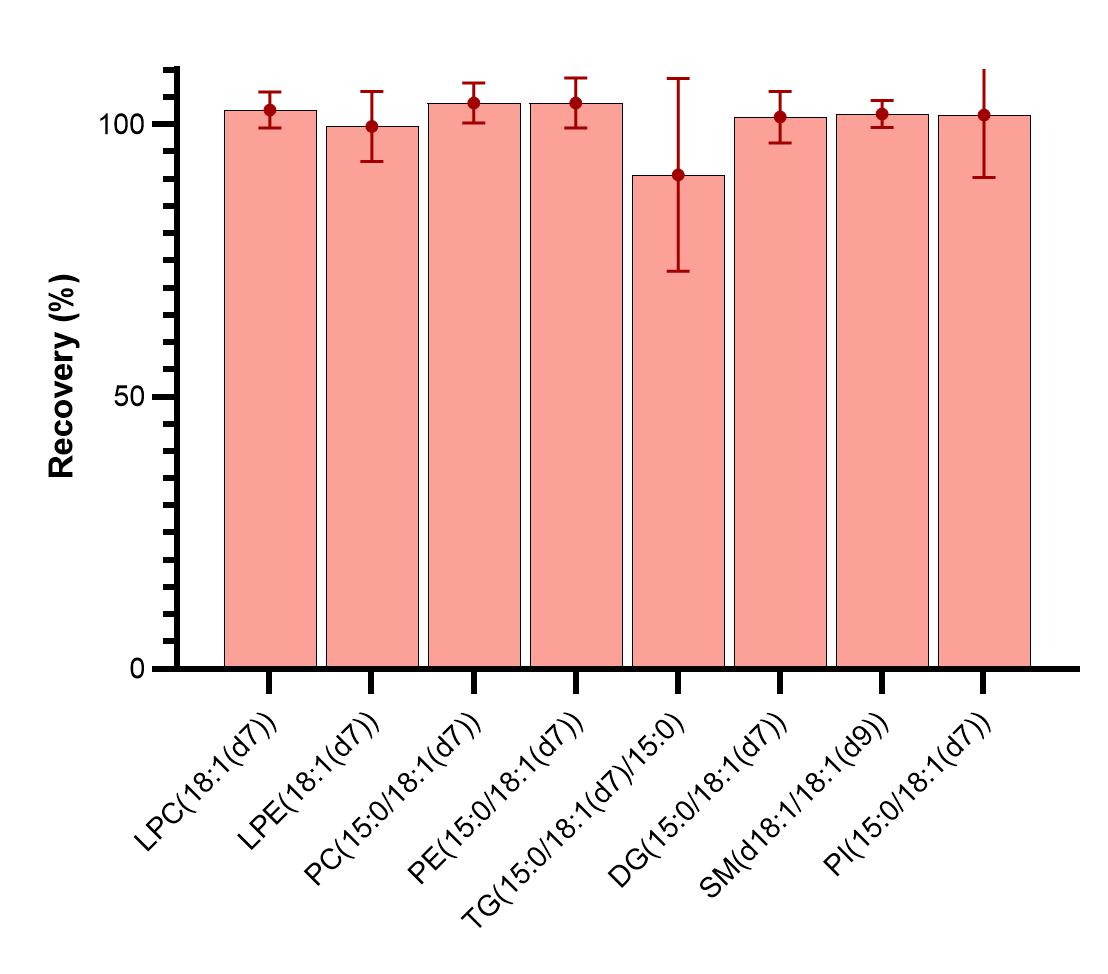


**Figure S7.** Recovery determination by comparing the signal response of the SPLASH^®^ LIPIDOMIX^®^ spiked into SRM 1950 aliquots, both before and after lipid extraction at three different concentrations –low, medium and high–. Plotted bars indicate the standard deviations.


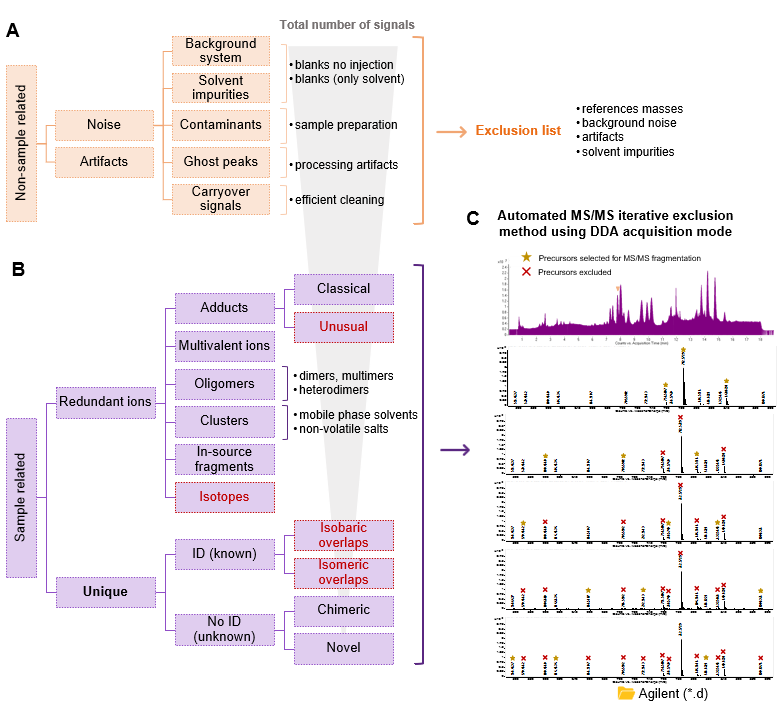


**Figure S8**. Classification of the multitude interferences and redundant information observed during RPLC-ESI-QTOF-MS analysis that can result in false positive annotations. **(A)** MS/MS datasets can yield spectra of poor quality due to **non-sample related interferences** from solvent masses and background system which could be resolved using background subtraction from a blank sample (only solvent) o blanks no injection and could be solved using exclusion lists for iterative MS/MS. **(B)** MS/MS datasets can also yield spectra not only due to poor quality low signal-to-noise and suboptimal collision energies but also due to **sample related interferences** from solvent-based clusters, oligomers, multiple adducts for the same lipids, isotope peaks, and/or unintended fragment ions and chimeric MS/MS spectra due to highly convoluted spectra (13). **(C)** Automated MS/MS iterative exclusion method with data-dependent acquisition (DDA) mode using five sequential injections (at 20 eV and 40 eV).

**Note:** It is important to consider the software-based annotation as a support for identification but not as an accurate analysis, which means that a manual inspection of the spectra by the researcher will always be necessary to corroborate or discard the annotation given by the software.

**Figure S9.** Example of false-positive annotation given by Lipid Annotator due to the unusual adduct formation ([M+HCOO]^-^) in a PC when formic acid is not used in mobile phases. Lipid Annotator identified *m/z* 826.563 as PC(35:4). After MS/MS data inspection, the neutral loss of formic acid from the precursor ion together with the formic acid fragment and the fatty acyl chains of PC(16:0/20:4) (PC 36:4) determined it as a false annotation.

**Figure S10.** All detected types of isobaric/isomeric overlaps classified according to Lipid Standard Initiative (14). **(A)** Types of isobaric overlaps, solved in all cases at MS1 level. **(B)** Types of isomeric overlaps solved at MS/MS level. Acyl chain length, functional group and *sn*-position isomers are solved with conventional CID-tandem MS strategies, while double bond position and functional group position isomers require advanced tandem MS strategies for resolution.


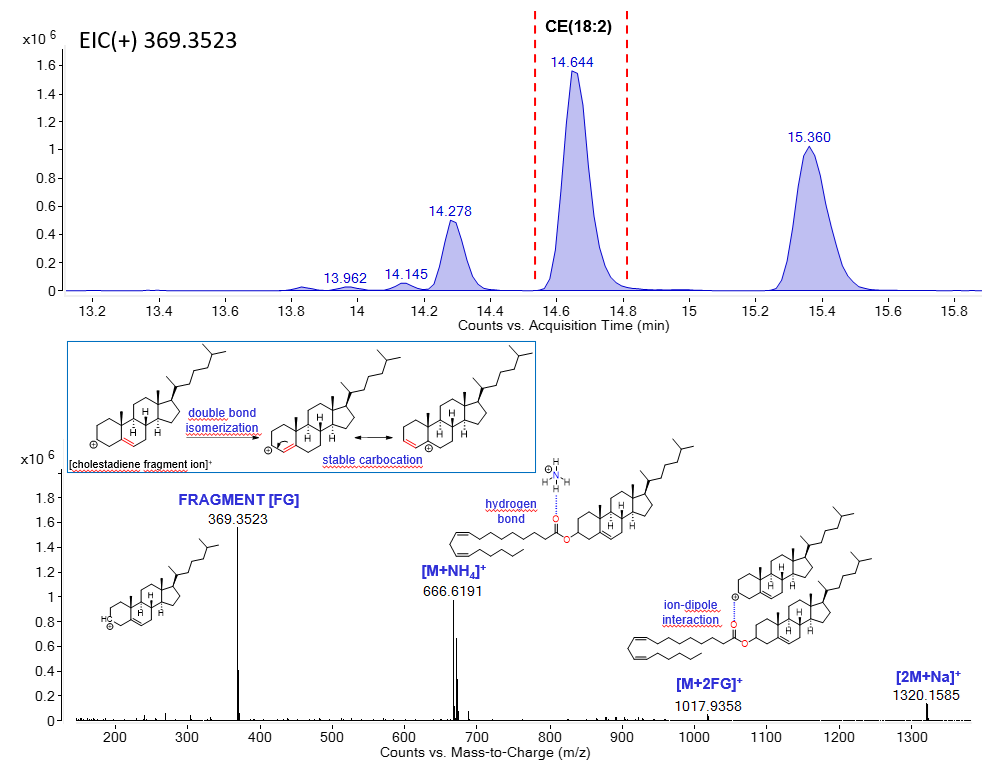


**Figure S11**. Example of in-source fragmentation in CE(18:2) observed during manual inspection.

**Figure S12**. **Isobaric overlap** examples observed during manual MS1 inspection: **(A)** **Type II isotopic overlap** occurs in all double bond series, that is, within a lipid class, the M+2-isotopologue of an unsaturated species overlaps M+0 of species with one double bond less (effect illustrated for sphingomyelins). **(B)** **Type II isotopic overlap** and **various adduct ions isobars** within lipid classes (effect illustrated for lysoglycerophospholipids).

**Figure S13**. Selected **fatty acyl chain length isomeric overlap** observed in diacyl glycerophospholipids containing unsaturated fatty acids during manual inspection. Example illustrated with the annotation of PC(36:3) = PC(18:1/18:2), RT 8.14 min and PC(16:0/20:3), RT 8.44 min, annotation based on both chromatographic retention time shifts and the intensity of the two fatty acyl anion fragment ratios observed in MS/MS (ESI(–)).

**Figure S14**. Selected **functional group isomeric overlap** observed during MS/MS manual inspection. Example illustrated for LPC(15:0/0:0), RT 2.19 min and LPE(18:0/0:0), RT 3.57 min based on specific diagnostic fragments and neutral losses of the glycerophospholipid head groups. Fragments 104.1058 and 184.0747 in LPC corresponding to the choline head group, and specific diagnostic neutral loss of 141.0191 in LPE

**Figure S15**. Selected ***sn*-position isomeric overlap** observed in monoacyl glycerophosphocholines containing saturated or unsaturated fatty acids during MS/MS manual inspection. Example illustrated with the annotation of LPC(16:0) = LPC(0:0/16:0), RT = 2.42 min and LPC(16:0/0:0), RT = 2.61 min) based on the intensity ratio of the phosphocholine fragment (184.0736) and the choline fragment (104.1070) observed in MS/MS.

**Figure S16**. Selected ***sn*-position isomeric overlap** observed in diacyl glycerophospholipids containing saturated fatty acids during MS/MS manual inspection. *sn*-isomeric diacyl GP are commonly present in SRM 1950 in a wide dynamic range but only those containing saturated fatty acids present complete co-elution under RP-UHPLC conditions. In these cases, the determination of fatty acyl chain composition is solely based in ESI(–)-CID-MS/MS experiments, where convoluted spectra are searched for diagnostic fragments of fatty acyl ion combinations compatible with the sum composition.

Assessment of the *sn*-position was performed according to Han et al (30), as for each pair of fatty acyl chain ions in the convoluted spectra, the ion corresponding to the fatty acyl chain bound to the *sn*-2 position is proven to have a higher relative intensity than that of the *sn*-1 position.

**(A)** Example illustrated with the annotation of PC(34:0). Fatty acyl anion fragments confirm the presence of FA(16:0) and FA(18:0), rejecting the annotations containing FA(14:0) and FA(20:0). The intensity ratio difference between the fatty acyl anion fragments allows the determination of the *sn*-position. Therefore, this lipid specie is annotated as PC(18:0/16:0). **(B)** Example illustrated with PC(30:0). Fatty acyl anion fragments confirm the presence of FA(14:0) and FA(16:0), rejecting the annotations containing FA(12:0) and FA(18:0). The intensity ratio difference between the fatty acyl anion fragments allows the determination of the *sn*-position. Therefore, this lipid specie is annotated as PC(16:0/14:0).

**Figure S17.** Kendrick mass defect (KMD) *vs* RT plot showing lipid series in SRM 1950 and mobile phase composition over the chromatographic separation method –mobile phases composition: I = isocratic elution**;** G = gradient elution– grouping lipid subclasses.

| **A**  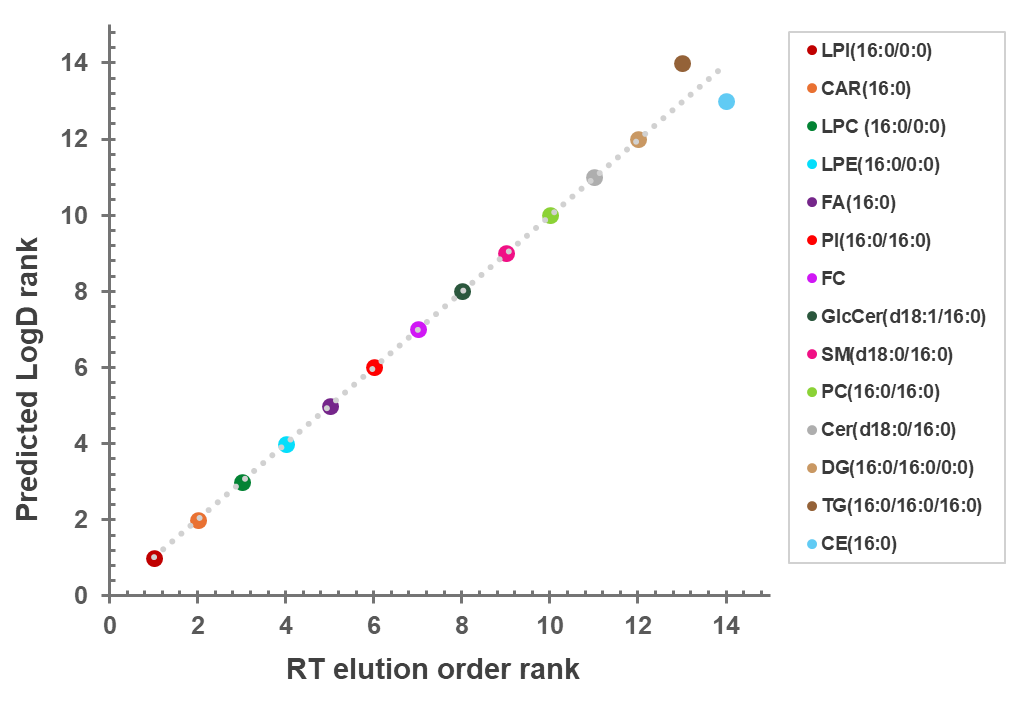  **B** |
| --- |
| 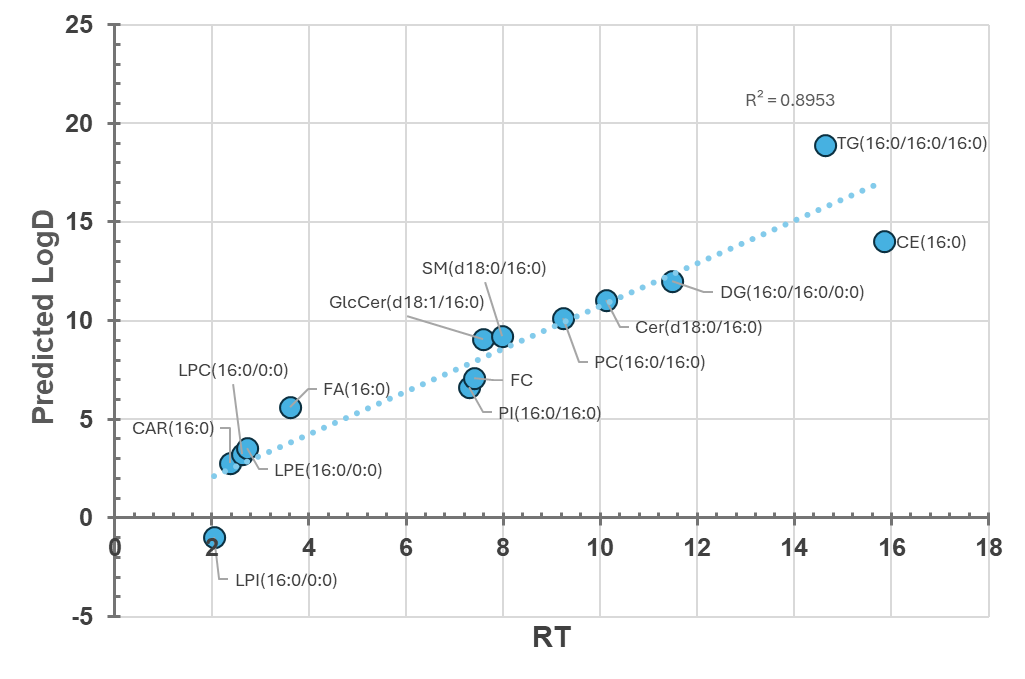 |

**Figure S18**. Representation of the correlation between retention time and LogD values predicted by Chemicalize for representative lipids bearing identical C16:0 FAC. Correlations were performed between ranks **(A)** and absolute values **(B)**.


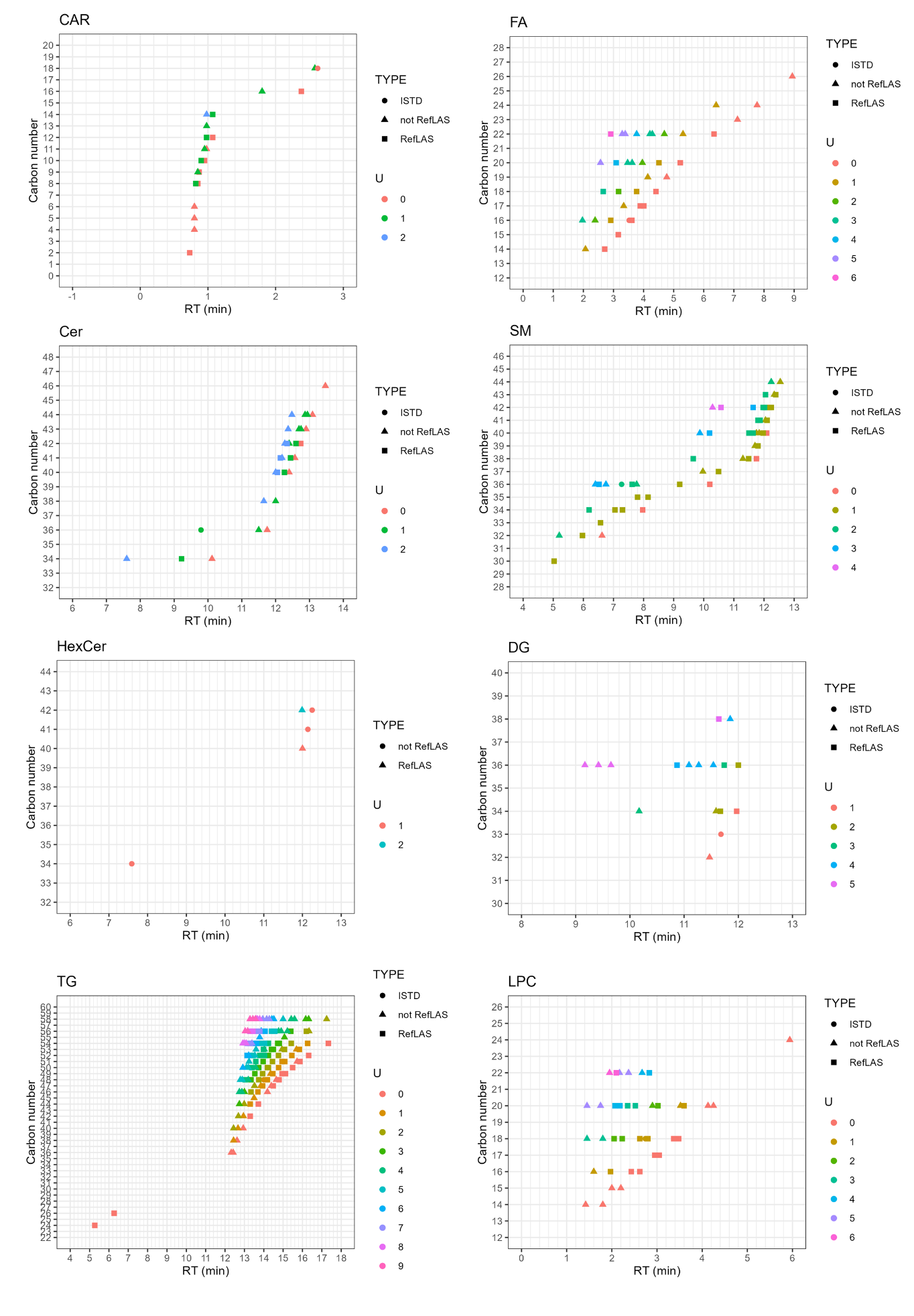


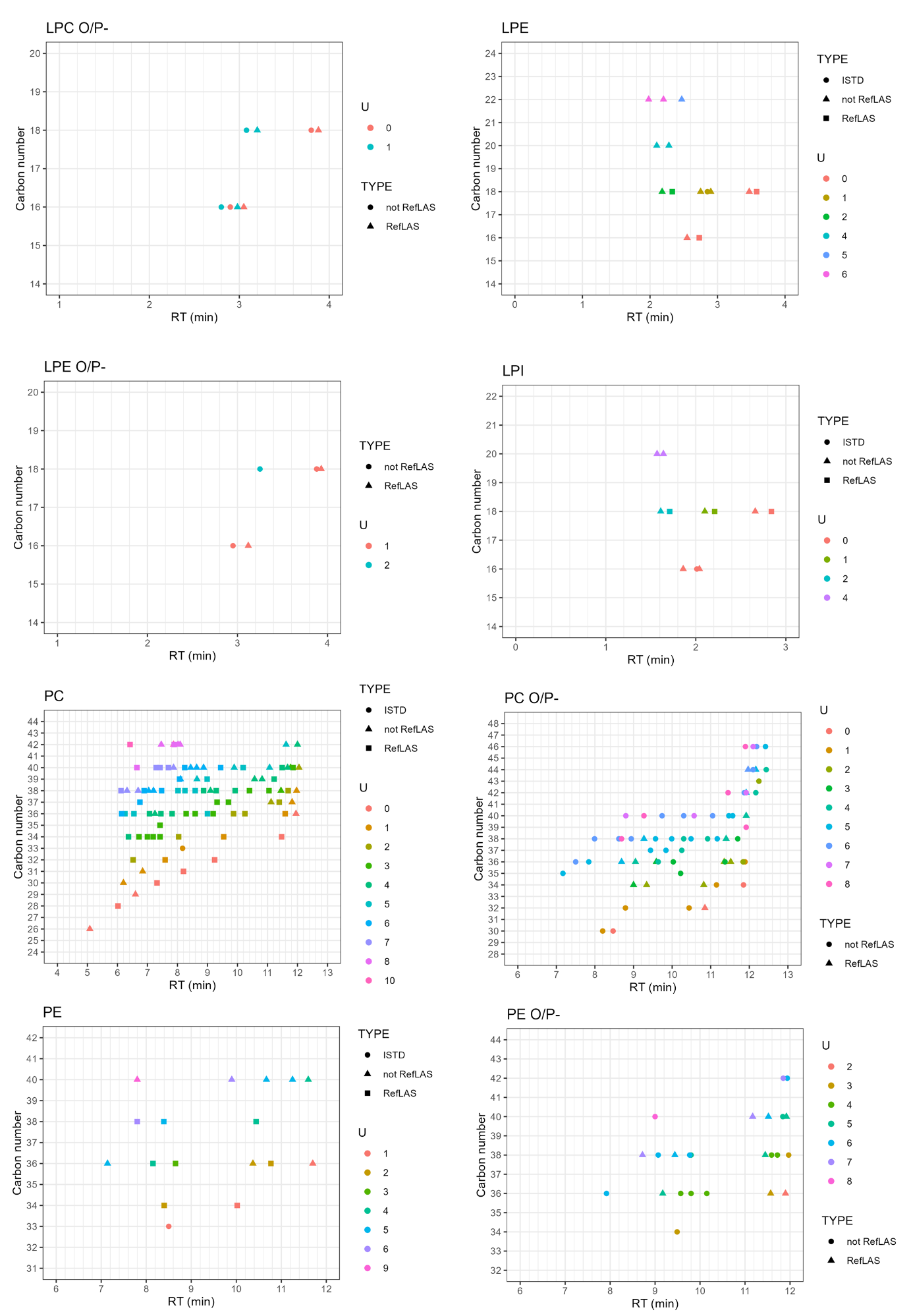


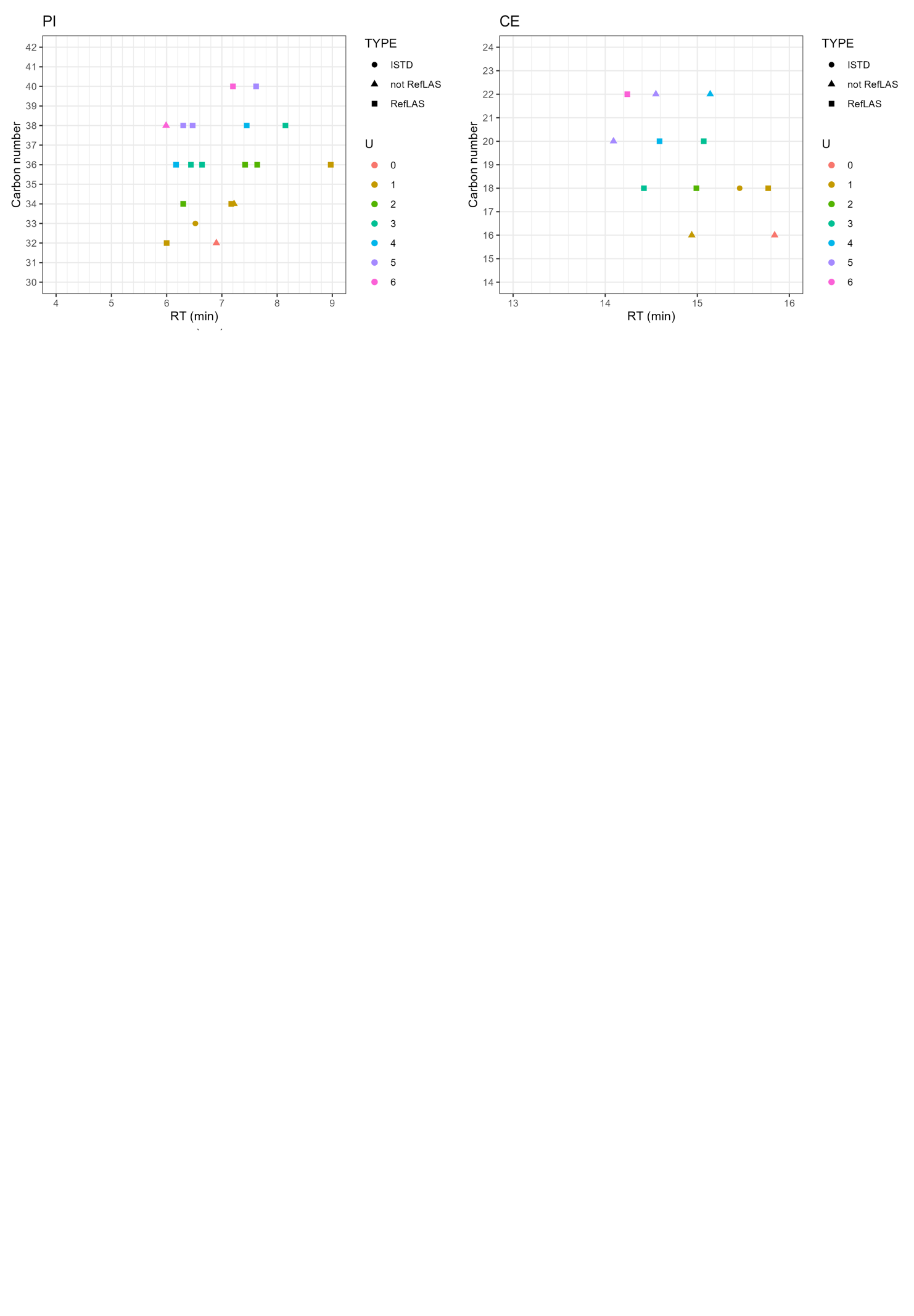


**Figure S19**. RT mapping –among all lipid subclasses detected– for lipid elution order assessment in ESI(+/–) of the sum composition.

ISTD: internal standard; RefLAS: Reference Lipid Annotation Set; RT: retention time; U: unsaturation number.

**Figure S20.** RT uncertainty according to the confidence interval (CI_95%_) (CI_95%, max_ -CI_95%, min_)) for each lipid species annotated in NIST SRM 1950 considering the 5 technical replicates of the sample. To determine the standard deviation and CI, the replicates of the polarity in which better ionize each lipid subclass were used, following the same criteria as described in the manuscript for the TFE algorithm of Profinder –RT was assed in ESI(–) in Cer, FA, LPI and PI lipid subclasses, and in ESI(+) in CAR, CE, DG, HexCer, LPC, LPE O/P, LPE, LPE O/P, PC, PC O/P, PE, PE O/P, SM and TG lipid subclasses–.


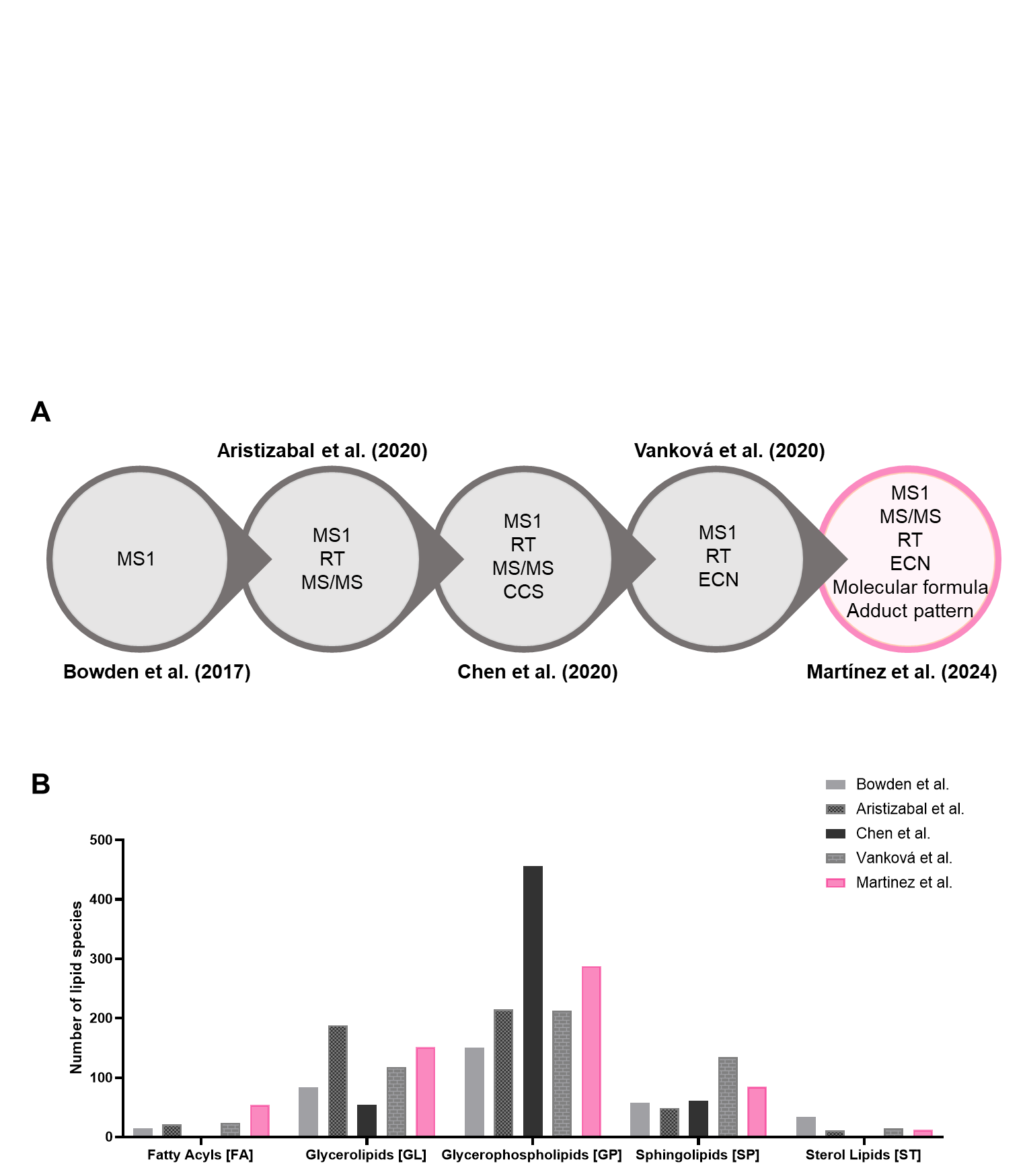


**Figure S21.** Comparison of the lipid database with already reported results. **(A)** Comparison of orthogonal information used for lipid annotation between already reported NIST SRM 1950 lipidomics studies and the current study. **(B)** Comparison of number of lipid species identified in each lipid subclass between already reported NIST SRM 1950 lipidomics studies and the current study.

**REFERENCES**

1. 1950.pdf.

https://tsapps.nist.gov/srmext/certificates/1950.pdf.

2. Pellegrino, R. M., A. Di Veroli, A. Valeri, L. Goracci, and G. Cruciani. 2014. LC/MS lipid profiling from human serum: a new method for global lipid extraction. *Anal Bioanal Chem.* **406**: 7937–7948.

3. Piédrola, I., S. Martínez, A. Gradillas, A. Villaseñor, V. Alonso-Herranz, I. Sánchez-Vera, E. Escudero, I. A. Martín-Antoniano, J. F. Varona, A. Ruiz, J. M. Castellano, Ú. Muñoz, and M. C. Sádaba. 2023. Deficiency in the production of antibodies to lipids correlates with increased lipid metabolism in severe COVID-19 patients. *Front Immunol.* **14**: 1188786.

4. Martínez, S., O. E. Albóniga, M. R. López-Huertas, A. Gradillas, and C. Barbas. 2024. Reinforcing the Evidence of Mitochondrial Dysfunction in Long COVID Patients Using a Multiplatform Mass Spectrometry-Based Metabolomics Approach. *J Proteome Res.* **23**: 3025-3040.

5. Koelmel, J. P., N. M. Kroeger, E. L. Gill, C. Z. Ulmer, J. A. Bowden, R. E. Patterson, R. A. Yost, and T. J. Garrett. 2017. Expanding Lipidome Coverage Using LC-MS/MS Data-Dependent Acquisition with Automated Exclusion List Generation. *J. Am. Soc. Mass Spectrom.* **28**: 908–917.

6. Defossez, E., J. Bourquin, S. von Reuss, S. Rasmann, and G. Glauser. 2023. Eight key rules for successful data-dependent acquisition in mass spectrometry-based metabolomics. *Mass Spectrom Rev.* **42**: 131–143.

7. Kirwan, J. A., H. Gika, R. D. Beger, D. Bearden, W. B. Dunn, R. Goodacre, G. Theodoridis, M. Witting, L.-R. Yu, I. D. Wilson, and metabolomics Quality Assurance and Quality Control Consortium (mQACC). 2022. Quality assurance and quality control reporting in untargeted metabolic phenotyping: mQACC recommendations for analytical quality management. *Metabolomics Off. J. Metabolomic Soc.* **18**: 70.

8. Lange, M., G. Angelidou, Z. Ni, A. Criscuolo, J. Schiller, M. Blüher, and M. Fedorova. 2021. AdipoAtlas: A reference lipidome for human white adipose tissue. *Cell Rep. Med.* **2**: 100407.

9. Koelmel, J. P., X. Li, S. M. Stow, M. J. Sartain, A. Murali, R. Kemperman, H. Tsugawa, M. Takahashi, V. Vasiliou, J. A. Bowden, R. A. Yost, T. J. Garrett, and N. Kitagawa. 2020. Lipid Annotator: Towards Accurate Annotation in Non-Targeted Liquid Chromatography High-Resolution Tandem Mass Spectrometry (LC-HRMS/MS) Lipidomics Using a Rapid and User-Friendly Software. *Metabolites*. **10**: 101.

10. Tsugawa, H., K. Ikeda, M. Takahashi, A. Satoh, Y. Mori, H. Uchino, N. Okahashi, Y. Yamada, I. Tada, P. Bonini, Y. Higashi, Y. Okazaki, Z. Zhou, Z.-J. Zhu, J. Koelmel, T. Cajka, O. Fiehn, K. Saito, M. Arita, and M. Arita. 2020. A lipidome atlas in MS-DIAL 4. *Nat Biotechnol.* **38**: 1159–1163.

11. Ni, Z., G. Angelidou, M. Lange, R. Hoffmann, and M. Fedorova. 2017. LipidHunter Identifies Phospholipids by High-Throughput Processing of LC-MS and Shotgun Lipidomics Datasets. *Anal Chem.* **89**: 8800–8807.

12. Bonney, J. R., and B. M. Prentice. 2021. Perspective on Emerging Mass Spectrometry Technologies for Comprehensive Lipid Structural Elucidation. *Anal Chem.* **93**: 6311–6322.

13. Stancliffe, E., M. Schwaiger-Haber, M. Sindelar, and G. J. Patti. 2021. DecoID improves identification rates in metabolomics through database-assisted MS/MS deconvolution. *Nat Methods*. **18**: 779–787.

14. Fiehn, O., D. Robertson, J. Griffin, M. van der Werf, B. Nikolau, N. Morrison, L. W. Sumner, R. Goodacre, N. W. Hardy, C. Taylor, J. Fostel, B. Kristal, R. Kaddurah-Daouk, P. Mendes, B. van Ommen, J. C. Lindon, and S.-A. Sansone. 2007. The metabolomics standards initiative (MSI). *Metabolomics*. **3**: 175–178.

15. Alcoriza-Balaguer, M. I., J. C. García-Cañaveras, F. J. Ripoll-Esteve, M. Roca, and A. Lahoz. 2022. LipidMS 3.0: an R-package and a web-based tool for LC-MS/MS data processing and lipid annotation. *Bioinformatics*. **38**: 4826-4828.

16. Köfeler, H. C., R. Ahrends, E. S. Baker, K. Ekroos, X. Han, N. Hoffmann, M. Holčapek, M. R. Wenk, and G. Liebisch. 2021. Recommendations for good practice in MS-based lipidomics. *J Lipid Res.* **62**: 100138.

17. Han, X., and R. W. Gross. 2001. Quantitative Analysis and Molecular Species Fingerprinting of Triacylglyceride Molecular Species Directly from Lipid Extracts of Biological Samples by Electrospray Ionization Tandem Mass Spectrometry. *Anal Biochem.* **295**: 88–100.

18. Koelmel, J. P., C. Z. Ulmer, C. M. Jones, R. A. Yost, and J. A. Bowden. 2017. Common cases of improper lipid annotation using high-resolution tandem mass spectrometry data and corresponding limitations in biological interpretation. *Biochim. Biophys. Acta Mol Cell Biol Lipids*. **1862**: 766–770.

19. Gonzalez-Riano, C., A. Gradillas, and C. Barbas. 2021. Exploiting the formation of adducts in mobile phases with ammonium fluoride for the enhancement of annotation in liquid chromatography-high resolution mass spectrometry based lipidomics. *J Chromatogr Open*. **1**: 100018.

20. Prabhu, G. R. D., E. R. Williams, M. Wilm, and P. L. Urban. 2023. Mass spectrometry using electrospray ionization. *Nat Rev Methods Primer*. **3**: 1–22.

21. Hu, C., W. Luo, J. Xu, and X. Han. 2022. RECOGNITION AND AVOIDANCE OF ION SOURCE-GENERATED ARTIFACTS IN LIPIDOMICS ANALYSIS. *Mass Spectrom. Rev.* **41**: 15–31.

22. Gathungu, R. M., P. Larrea, M. J. Sniatynski, V. R. Marur, J. A. Bowden, J. P. Koelmel, P. Starke-Reed, V. S. Hubbard, and B. S. Kristal. 2018. Optimization of Electrospray Ionization Source Parameters for Lipidomics To Reduce Misannotation of In-Source Fragments as Precursor Ions. *Anal Chem.* **90**: 13523–13532.

23. Chen, X., Y. Yin, Z. Zhou, T. Li, and Z.-J. Zhu. 2020. Development of a combined strategy for accurate lipid structural identification and quantification in ion-mobility mass spectrometry based untargeted lipidomics. *Anal Chim Acta*. **1136**: 115–124.

24. Bowden, J. A., A. Heckert, C. Z. Ulmer, C. M. Jones, J. P. Koelmel, L. Abdullah, L. Ahonen, Y. Alnouti, A. M. Armando, J. M. Asara, T. Bamba, J. R. Barr, J. Bergquist, C. H. Borchers, J. Brandsma, S. B. Breitkopf, T. Cajka, A. Cazenave-Gassiot, A. Checa, M. A. Cinel, R. A. Colas, S. Cremers, E. A. Dennis, J. E. Evans, A. Fauland, O. Fiehn, M. S. Gardner, T. J. Garrett, K. H. Gotlinger, J. Han, Y. Huang, A. H. Neo, T. Hyötyläinen, Y. Izumi, H. Jiang, H. Jiang, J. Jiang, M. Kachman, R. Kiyonami, K. Klavins, C. Klose, H. C. Köfeler, J. Kolmert, T. Koal, G. Koster, Z. Kuklenyik, I. J. Kurland, M. Leadley, K. Lin, K. R. Maddipati, et al. 2017. Harmonizing lipidomics: NIST interlaboratory comparison exercise for lipidomics using SRM 1950–Metabolites in Frozen Human Plasma. *J Lipid Res.* **58**: 2275–2288.

25. Aristizabal-Henao, J. J., C. M. Jones, K. A. Lippa, and J. A. Bowden. 2020. Nontargeted lipidomics of novel human plasma reference materials: hypertriglyceridemic, diabetic, and African-American. *Anal Bioanal Chem.* **412**: 7373–7380.

26. Vaňková, Z., O. Peterka, M. Chocholoušková, D. Wolrab, R. Jirásko, and M. Holčapek. 2022. Retention dependences support highly confident identification of lipid species in human plasma by reversed-phase UHPLC/MS. *Anal Bioanal Chem.* **414**: 319–331.

27. Fiehn, O., D. Robertson, J. Griffin, M. van der Werf, B. Nikolau, N. Morrison, L. W. Sumner, R. Goodacre, N. W. Hardy, C. Taylor, J. Fostel, B. Kristal, R. Kaddurah-Daouk, P. Mendes, B. van Ommen, J. C. Lindon, and S.-A. Sansone. 2007. The metabolomics standards initiative (MSI). *Metabolomics*. **3**: 175–178.

28. Ovčačíková, M., M. Lísa, E. Cífková, and M. Holčapek. 2016. Retention behavior of lipids in reversed-phase ultrahigh-performance liquid chromatography-electrospray ionization mass spectrometry. *J Chromatogr. A*. **1450**: 76–85.

29. Bishop, L. M., T. Shen, and O. Fiehn. 2023. Improving Quantitative Accuracy in Nontargeted Lipidomics by Evaluating Adduct Formation. *Anal Chem.* **95**: 12683–12690.

30. Han, X. 2016. Lipidomics: comprehensive mass spectrometry of lipids. *Wiley*.
